# Transient Middle Cerebral Artery Occlusion in Rats as a Nonclinical Model of Ischemic Stroke: A Systematic Review

**DOI:** 10.3390/cimb48060632

**Published:** 2026-06-17

**Authors:** Priscila Mendes, Joana Pinto, Carole Mateus, Inês Guerra, Vanessa Mateus

**Affiliations:** 1 H&TRC-Health and Technology Research Center, ESTeSL-Escola Superior de Tecnologia da Saúde, Instituto Politécnico de Lisboa, 1990-096 Lisboa, Portugal; priscila.mendes@essl.ipl.pt (P.M.); 2021322@alunos.essl.ipl.pt (C.M.); 2021346@alunos.essl.ipl.pt (I.G.); 2 Research Institute for Medicines (iMed.ULisboa), Faculty of Pharmacy, Universidade de Lisboa, Av. Professor Gama Pinto, 1649-003 Lisboa, Portugal; joanapinto7@edu.ulisboa.pt

**Keywords:** ischemic stroke, transient middle cerebral artery occlusion, tifMCAO, rats, experimental model, methodological variability, outcome assessment, reproducibility, translational relevance

## Abstract

Background: Ischemic stroke remains a leading cause of mortality and disability worldwide. Despite extensive preclinical research, most neuroprotective strategies have failed to translate into clinical benefit, partly due to methodological variability. The transient intraluminal filament middle cerebral artery occlusion (tifMCAO) model is widely used, yet its implementation lacks consistency. This review aimed to characterize tifMCAO methodologies in adult rats and examine how experimental variability relates to reported outcomes. Methods: A systematic review was conducted following PRISMA guidelines. Studies using tifMCAO in adult rats were included. MEDLINE (PubMed), Web of Science, and Scopus were searched up to March 2025. Risk of bias was assessed using the SYRCLE tool and reporting quality using the ARRIVE checklist. The protocol was registered in PROSPERO (CRD420251140869). Results were synthesized narratively. Results: A total of 125 studies were included. A commonly used framework involved male Sprague–Dawley rats (6–12 weeks), silicone-coated monofilaments, occlusion durations of 60–120 min (most frequently 90 min), and isoflurane anesthesia, although this reflects methodological convergence rather than true standardization. Substantial variability was observed across methodological parameters. Variations in ischemia duration, filament properties, and anesthesia were associated with differences in infarct size, blood–brain barrier disruption, and functional outcomes. Conclusions: The tifMCAO model shows partial methodological convergence alongside significant variability influencing outcomes. Improved standardization and reporting are essential to enhance reproducibility and translational relevance.

## 1. Introduction

Ischemic stroke remains one of the leading causes of mortality and long-term disability worldwide, imposing a substantial global health and socioeconomic burden [1]. Despite major advances in acute stroke management, current therapeutic options remain limited to a narrow subset of patients. Currently, intravenous thrombolysis with recombinant tissue plasminogen activators and mechanical thrombectomy constitute the standard reperfusion therapies, with proven clinical efficacy, when administered within a narrow therapeutic window, typically 4.5 to 6 h after symptom onset. These therapies, depending on early diagnosis, are usually achievable only in specialized stroke centers [2,3]. Therefore, the development of novel therapeutic strategies based on neuroprotection and post-stroke recovery is crucial.

Numerous compounds that showed high efficacy in animal studies have failed to translate into clinical benefits [4,5]. This persistent translational gap is a critical limitation in preclinical stroke research due to the lack of methodological standardization and reproducibility across experimental models.

The transient intraluminal filament middle cerebral artery occlusion (tifMCAO) model in rats has become the most widely used to investigate the mechanisms of neuronal injury, inflammation, and neuroprotection, as well as to evaluate potential therapeutic interventions [6]. This model enables the induction of transient ischemia by inserting a filament into the internal carotid artery to occlude the origin of the middle cerebral artery [7,8]. Nonetheless, key parameters, including animal strain and age, anesthesia, temperature control, filament diameter and coating, occlusion duration, and reperfusion timing, can significantly influence infarct volume, neurological outcomes, and mortality [9,10,11]. The inconsistent reporting or control of these variables restrains reliable comparisons across studies and limits the translational value of preclinical findings. Given the central role of the tifMCAO model in experimental stroke research and the wide variation in its implementation, a systematic and comprehensive synthesis of methodological approaches is needed. Therefore, this systematic review aims to provide a structured overview of the tifMCAO model in adult rats, focusing on the techniques used and the variability in outcome assessment. Specifically, it seeks to identify and systematize the main surgical and procedural approaches used to induce transient ischemia, and to characterize the range of reported outcomes, including infarct size, brain edema, blood–brain barrier disruption, and related functional and molecular markers. By integrating methodological and outcome-related data, this review aims to improve comparability across studies, enhance reproducibility, and support greater standardization in preclinical stroke research.

## 2. Materials and Methods

This systematic review was conducted in accordance with the Preferred Reporting Items for Systematic Review and Meta-Analysis (PRISMA 2020) guidelines [12].

### 2.1. Protocol Registration

The protocol for this systematic review is publicly available on the Open Science Framework (OSF) platform (10.17605/OSF.IO/F4EPQ) and registered in the PROSPERO (CRD420251140869). Additional data is available from the authors upon request.

### 2.2. Eligibility Criteria

This systematic review applies to an adapted PICO+D framework for experimental in vivo nonclinical research. The population consists of adult rats, and the intervention is the induction of the transient intraluminal filament middle cerebral artery occlusion (tifMCAO) model. Given the methodological heterogeneity of preclinical studies, no formal comparator was defined; instead, variations in techniques used to induce tifMCAO were systematically analyzed. The outcomes assessed include infarct size, brain edema, blood–brain barrier disruption, molecular and biochemical biomarkers in brain and peripheral samples, neurobehavioral and cognitive outcomes, physiological parameters (e.g., body weight), and mortality. The study design comprises in vivo experimental studies. Studies selected according to the following inclusion criteria: only studies that applied the transient intraluminal filament middle cerebral artery occlusion (tifMCAO) model as the primary intervention to induce cerebral ischemia were considered. Studies using adult rats were included, without restrictions regarding strain or body weight. No language restrictions were applied.

Studies were excluded if they involved animals with comorbidities or were conducted using ex vivo, in vitro, or in silico models. In addition, studies published before 2018 were excluded to focus on recent methodological developments following the latest comprehensive review of the tifMCAO model.

Accordingly, this review aims to address the following question: What techniques are used to induce transient ischemia using the tifMCAO model in adult rats, and how do variations in experimental methodology relate to differences in reported outcomes?

### 2.3. Information Sources and Search Strategy

MEDLINE (PubMed), Web of Science, and Scopus were the biomedical electronic databases used for a highly sensitive search strategy. A comprehensive approach was employed, using descriptors related to the four key terms, including middle cerebral artery occlusion, intraluminal filament method, animal models, and rat, along with their synonyms. These terms were combined using the Boolean operators “AND” and “OR” to identify and select eligible studies. The search strategy, adapted for each biomedical electronic database, is available in the Supplemental Materials (Table S1).

### 2.4. Selection Process

After applying the search strategy across each database, retrieved articles were exported to the Systematic Reviews Web Application Rayyan (Rayyan Systems Inc., Cambridge, MA, USA) [13]. Duplicates were removed, and two independent reviewers screened titles and abstracts according to the predefined inclusion and exclusion criteria. The same reviewers then assessed full texts to determine their eligibility. In cases of disagreement between the two reviewers at any stage, a third reviewer provided a final decision. The study selection process was documented and summarized using the PRISMA flowchart [12].

### 2.5. Data Collection Process

Data from the included studies were extracted independently by two reviewers using Microsoft Excel (Microsoft Corporation, Redmond, WA, USA). A standardized data extraction sheet was developed and pilot-tested to ensure consistency. The extracted variables are detailed in Section 2.6. Data was extracted from the text, figures, and/or tables. Discrepancies between reviewers were resolved through discussion, with a third reviewer when necessary, to ensure accuracy and consistency. Given the methodological focus of this review, no attempts were made to contact study authors for additional information. Studies in Chinese and Russian were translated using automated translation tools, primarily DeepL Translator (DeepL SE, Cologne, Germany).

### 2.6. Data Items

The registered data was:

• Study Design: Parameters related to the experimental in vivo nonclinical study (e.g., sample size, experimental design, and follow-up period/post-occlusion assessment time).
• Population: Rat-related parameters, including strain, age, sex, and body weight.
• Intervention: tifMCAO-related parameters, including type of filament, filament diameter and length, method of insertion, duration of occlusion, temperature control during the procedure, use and type of anesthesia and analgesia, and reperfusion period.
• Outcomes: Infarct size, brain edema, blood–brain barrier disruption, molecular and biochemical biomarkers in brain and peripheral samples, neurobehavioral and cognitive outcomes, physiological parameters (e.g., body weight), and mortality.
• Study Identification: Study identification details, including authors, article title, and year of publication.

In addition to absolute frequencies (*n*), a qualitative classification of frequency was applied to facilitate the interpretation of commonly used experimental parameters. Categories were defined a priori based on the number of studies reporting each parameter as follows: very high (*n* ≥ 30), high (*n* = 15–29), moderate (*n* = 5–14), and low (*n* < 5), considering the total number of included studies (*n* = 125). This classification was applied consistently across all tables summarizing methodological variables and was intended as a descriptive aid, rather than being derived from statistical power calculations or distributional assumptions.

### 2.7. Risk of Bias and Reporting Quality Assessment

The risk of bias was assessed using the Systematic Review Centre for Laboratory Animal Experimentation (SYRCLE) tool, with two reviewers independently classifying each study as low (green), moderate (yellow), or high (red) risk of bias [14]. Robvis (Risk-of-bias visualization) tool was used for data representation [15]. Reporting quality, as a proxy for internal validity, was independently evaluated by the same two reviewers using the Animal Research: Reporting of In Vivo Experiments (ARRIVE) checklist, scored by domain (high = 2, moderate = 1, low = 0). Discrepancies were solved through a third reviewer [16]. Study-level risk-of-bias and reported quality assessments are available from the authors upon request.

### 2.8. Data Synthesis

Given the substantial heterogeneity in experimental design, methodological parameters, and outcome measures across the included studies, a structured narrative synthesis was conducted in accordance with the Synthesis Without Meta-analysis (SWiM) guidelines [17]. Studies were grouped according to key methodological domains and outcome categories, including: (1) ischemia induction parameters (e.g., occlusion duration, filament characteristics); (2) physiological monitoring and perioperative management; and (3) outcome assessment, encompassing structural (infarct size, edema), vascular (blood–brain barrier disruption), molecular, and functional endpoints. Within each domain, studies were comparatively appraised to identify consistent patterns, sources of variability, and methodological factors associated with differences in reported outcomes. Key characteristics and findings were systematically tabulated to facilitate cross-study comparison and enhance interpretability.

To ensure reliability, the data extraction was independently coded by two reviewers. Any discrepancies were cross-checked. In the event of disagreement, a consensus was reached, or a third reviewer intervened to resolve the issue. The agreement level between the two reviewers was quantified using the kappa coefficient.

### 2.9. Use of Generative Artificial Intelligence (GenAI)

Generative artificial intelligence tools were used to assist in language refinement, text structuring, and clarity improvement during manuscript preparation. These tools were not used for study design, data collection, data extraction, analysis, or interpretation. All scientific content, data synthesis, and conclusions were developed and validated by the authors.

## 3. Results

### 3.1. Study Selection

**Figure 1:**
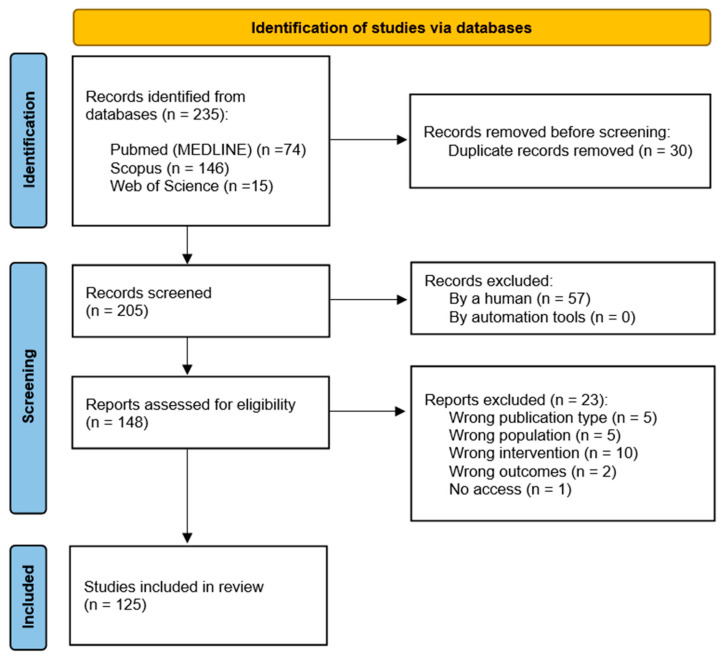
PRISMA flow diagram of the identification, screening, and inclusion strategy for the study selection [12].

The search strategy conducted across biomedical electronic platforms retrieved a total of 235 articles. After removing 30 duplicates, 205 articles remained for title and abstract screening. This initial screening led to the exclusion of 57 reports. The full texts of the remaining 148 potentially eligible studies were then assessed in detail according to predefined inclusion and exclusion criteria. Following this evaluation, 23 studies were excluded for the following reasons: the publication was a review article (*n* = 5); the animal used was not a rat or was younger than six weeks of age (*n* = 5); the exposure did not involve tifMCAO (*n* = 10); studies that did not report any outcomes of interest (*n* = 2); and the full text was not accessible (*n* = 1). In total, 125 studies met all eligibility criteria and were included in the qualitative synthesis (Figure 1).

### 3.2. Study Characteristics

A total of 125 studies met the inclusion criteria and were included in the analysis [18,19,20,21,22,23,24,25,26,27,28,29,30,31,32,33,34,35,36,37,38,39,40,41,42,43,44,45,46,47,48,49,50,51,52,53,54,55,56,57,58,59,60,61,62,63,64,65,66,67,68,69,70,71,72,73,74,75,76,77,78,79,80,81,82,83,84,85,86,87,88,89,90,91,92,93,94,95,96,97,98,99,100,101,102,103,104,105,106,107,108,109,110,111,112,113,114,115,116,117,118,119,120,121,122,123,124,125,126,127,128,129,130,131,132,133,134,135,136,137,138,139,140,141,142]. All studies were experimental in vivo nonclinical investigations using rat tifMCAO models to induce transient cerebral ischemia. The included studies encompassed a wide range of experimental designs and follow-up periods, reflecting variability in the timing of outcome assessment across acute and chronic phases of ischemic injury. A broad spectrum of outcomes was reported, including structural, molecular, and functional parameters, such as infarct size, brain edema, blood–brain barrier disruption, biomarkers, and neurobehavioral outcomes.

### 3.3. Animal-Related Parameters

Animal-related parameters, including sample size, strain, sex, age, and body weight, were systematically extracted. A marked predominance of Sprague–Dawley rats was observed (*n* = 92), followed by Wistar (*n* = 27), Wistar–Kyoto (*n* = 2), and Lewis rats (*n* = 1). A small number of studies did not specify the strain (*n* = 5).

Most studies used male animals (*n* = 110), whereas female rats were included in a limited number of studies (*n* = 6). A few studies included both sexes in separate experimental groups (*n* = 3), while others did not report the sex of the animals (*n* = 6).

Regarding age, the most reported range was 6–12 weeks (*n* = 46). Several studies described animals as “adult” (*n* = 38) or “young adult” (*n* = 1) without specifying exact age, and a substantial number did not report age (*n* = 37). The use of aged animals (>18 months) was rare (*n* = 1).

Reported body weight ranged from 180 g to 641 g across studies. Detailed information on animal-related parameters is provided in the Supplementary Materials (Table S2).

### 3.4. tifMCAO Induction Parameters

The characteristics of ischemia induction in rat tifMCAO models (Table 1 and Table 2). Ischemia duration varied considerably across studies; however, protocols within the 60–120 min range were predominant. A duration of 90 min was the most frequently reported (*n* = 42), followed by 120 min (*n* = 40) and 60 min (*n* = 25). Less commonly used durations were rarely applied, including 75 min (*n* = 4), 30 min (*n* = 3), 100 min (*n* = 1), and ≥180 min (*n* = 2). A subset of studies employed variable occlusion durations (*n* = 4), and some did not report the duration (*n* = 4).

Filament characteristics also showed substantial variability. Silicone-coated nylon monofilaments were the predominant configuration (*n* = 50), typically with tip diameters of 0.35–0.40 mm and suture sizes ranging from 3-0 to 5-0. Filaments without coating but with tip modification, such as rounded or cylindrical tips, were also reported (*n* = 14), as were poly-L-lysine-coated filaments (*n* = 8). Other modifications were rare and included heparin-coated (*n* = 1), poly-L-lysine plus heparin-coated (*n* = 1), paraffin-coated (*n* = 1), polysiloxane (*n* = 1), fire-polished tip (*n* = 1), and Silicon–Teflon filaments combined with poly-L-lysine (*n* = 1). In addition, five studies did not report filament characteristics (*n* = 12), and eleven reported silicone-coated filaments without specifying the base material (*n* = 11). The remaining studies provided incomplete reporting of filament characteristics, including base material, coating or tip modification, and tip diameter or suture size. Adjustments of filament diameter and insertion depth according to animal body weight were reported in several studies as relevant methodological considerations for achieving consistent occlusion [22,63,88,95].

### 3.5. Anesthesia Protocols

Anesthesia protocols used in tifMCAO procedures are presented in Table 3. Isoflurane inhalation was the most used anesthetic (*n* = 49), typically administered at 3–5% for induction and 1–3% for maintenance. Chloral hydrate was also frequently used (*n* = 17), generally administered intraperitoneally at a dose of 300–400 mg/kg. Ketamine/xylazine combinations were reported in a moderate number of studies (*n* = 12), most commonly via intraperitoneal, intramuscular, or intravenous routes. Similarly, pentobarbital was used in a comparable number of studies (*n* = 12), typically administered at doses of 30–50 mg/kg.

Other anesthetic agents were less frequently reported, including halothane (*n* = 5), sevoflurane (*n* = 4), ketamine alone (*n* = 2), urethane (*n* = 2), enflurane (*n* = 1), propofol (*n* = 1), and chloroform (*n* = 1). One study reported the use of pentobarbital infusion, after the occlusion, instead of isoflurane due to the vasodilatory effects of isoflurane on cerebral circulation, and in preference to chloral hydrate to minimize its hypotensive effects [30]. A combination of medetomidine, midazolam, and fentanyl was reported in a single study [70]. Notably, a considerable number of studies did not report anesthesia protocols in detail (*n* = 11).

### 3.6. Infarct Size Outcomes

Infarct size outcomes and their methodological approaches are summarized in Table 4. Infarct size was assessed using macroscopy (2,3,5-triphenyltetrazolium chloride (TTC) staining) or histology, quantified by planimetric analysis, or assessed in vivo using MRI sequences (T2-weighted/DWI).

Relative infarct size normalized to the brain hemispheres (%) was frequently measured (*n* = 51), whereas absolute infarct volume (mm^3^) was reported in a subset of studies (*n* = 21). Several studies reported both absolute and relative infarct volume (*n* = 10). Edema-corrected infarct size, typically calculated using indirect methods such as the Swanson correction, was also frequently reported (*n* = 33) to reduce swelling-related bias. Several studies reported infarct size assessment without providing quantitative data (absolute or relative values) in the results.

Most studies assessed infarction approximately 24 h after MCAO, with additional evaluations conducted in the acute (3–72 h) and chronic (≥7 d) phases.

In addition to quantitative measures, some studies described lesion patterns based on anatomical distribution. Assessment of ischemic core and penumbra was reported (*n* = 3), primarily using MRI-based techniques (DWI/PWI). Infarction severity has been categorized into subcortical (mild) and hemispheric (severe) patterns, reflecting the extent of vascular compromise. Mild lesions are generally confined to subcortical regions, whereas hemispheric infarctions extend across both subcortical and cortical territories [32,118]. This spatial distribution has been associated with the occlusion duration and collateral circulation capacity [80,125].

According to the included studies, infarct size in control (untreated) groups showed substantial variability, reflecting differences in experimental conditions and model severity.

When expressed as a percentage of the ipsilateral hemisphere, infarct size varied widely. The lowest reported mean value in untreated MCAO groups was around 8%, corresponding to mild infarctions restricted primarily to subcortical regions [118]. Under standard experimental conditions, the minimum volumes in vehicle groups tend to be around 14% [109]. At the upper extreme, infarct volumes reached up to 78% of the ipsilateral hemisphere at 48 h post-reperfusion, reflecting the peak of the lesion and swelling [54]. Other severe models displayed values in the range of approximately 58–60% [95,121].

Similarly, when reported as absolute volume, mean infarct sizes ranged from approximately 40–50 mm^3^ in smaller lesions [29,32,45] to values exceeding 248 mm^3^ in more severe models [88]. In typical transient MCAO protocols, baseline infarct volumes in control groups commonly ranged between approximately 120–160 mm^3^ [107,109]. In some studies, employing more severe or prolonged occlusion conditions, graphical representations suggested infarct volumes approaching 500–600 mm^3^, highlighting the potential extent of ischemic damage in the absence of intervention.

Larger infarct volumes were generally associated with longer ischemia durations and optimized filament parameters, whereas shorter durations were often associated with smaller or more variable lesions [22,55,109].

In addition to methodological differences in infarct quantification, several experimental variables were associated with variability in infarct size across studies. Sex-related differences were reported, with hormonal status influencing lesion severity, particularly across different phases of the estrous cycle [22,39]. Age-related effects were less consistent, with one study reporting comparable infarct volumes between young adult and middle-aged male rats under similar experimental conditions [22].

Filament diameter was also reported as an important factor influencing infarct size, with its effects depending on animal characteristics such as body weight, age, and sex. Smaller filament diameters (e.g., 0.43–0.45 mm) were associated with more consistent infarct induction in heavier animals (>500 g), highlighting the need for protocol adjustment across experimental conditions [22].

Anesthetic protocols may also contribute to variability in infarct size, as one study reported the use of pentobarbital instead of isoflurane or chloral hydrate to minimize cerebrovascular and hemodynamic effects [30].

### 3.7. Brain Edema Assessment

Methods for assessing brain edema are summarized in Table 5. Brain water content, typically quantified using the wet/dry method, was the most frequently reported approach (*n* = 14), with measurements usually performed between 24 and 72 h post-MCAO. This method provides a global estimate of tissue hydration but lacks spatial resolution.

Cerebral edema was also assessed by means of hemispheric volume comparisons (*n* = 7), usually expressed as a percentage of volume change. MRI-based techniques, including T2-weighted imaging, apparent diffusion coefficient (ADC) mapping, and T1 mapping, were less frequently used (*n* = 2) but enabled spatial and longitudinal evaluation of edema progression [81,139].

In the included studies, cerebral edema values showed variability depending on the assessment method and model severity. When measured as brain water content, values in untreated MCAO groups typically ranged from approximately 80.0–82.9% [43,101,131]. Values exceeding 100% are not consistent with the standard wet/dry calculation of brain water content and may reflect differences in calculation methods or reporting across studies [51].

When expressed as hemispheric swelling, edema values ranged from approximately 7–9% in mild to values between ~14–22% in more severe MCAO models [37,118].

MRI-based assessments showed temporal changes in edema, with low diffusion and high T2 signal at 24 h indicating combined cytotoxic and vasogenic edema, followed by increased diffusion and persistent T2 hyperintensity up to 120 h, consistent with predominant vasogenic edema and blood–brain barrier disruption [139].

### 3.8. Blood–Brain Barrier Disruption

Blood–brain barrier (BBB) disruption outcomes are summarized in Table 6. BBB permeability was most assessed using Evans Blue extravasation (*n* = 11), based on intravenous dye injection followed by spectrophotometric quantification of tissue accumulation, typically expressed as µg/g tissue or optical density. This approach enables the evaluation of macromolecular leakage but is highly dependent on experimental parameters such as dye circulation time and extraction methodology.

Fluorescent tracer extravasation was also reported (*n* = 5), using compounds such as sodium fluorescein (NaFL), dextran, and albumin-based tracers to assess microvascular permeability. These methods provide sensitive detection of BBB disruption but are influenced by tracer size and molecular weight, limiting comparability across studies.

MRI-based techniques, including contrast-enhanced imaging using gadolinium agents, were applied less frequently (*n* = 3) but enabled in vivo and longitudinal assessment of BBB permeability through signal enhancement or permeability indices.

In addition to permeability assays, several studies have evaluated BBB integrity by analyzing tight junction proteins and matrix metalloproteinases (*n* = 9), including claudin-5, occludin, and zonula occludens-1 (ZO-1), as well as matrix metalloproteinases MMP-2 and MMP-9. These markers reflect molecular alterations associated with barrier disruption but do not directly quantify permeability.

Direct assessment of BBB transport and permeability kinetics using in situ brain perfusion with radiolabeled tracers was rarely reported (*n* = 1), likely due to its technical complexity and requirement for strict physiological control [100].

Overall, BBB disruption was predominantly assessed within the acute phase (6–48 h post-MCAO), with consistent evidence of increased permeability across methods, including Evans Blue, fluorescent tracers, and radiolabeled assays, confirming marked barrier breakdown following ischemic injury [26,37,43,48,100,101,110,121,131,138,141].

### 3.9. Molecular and Peripheral Biomarkers

Molecular and peripheral biomarkers assessed in tifMCAO studies are summarized in Table 7. In brain tissue, a wide range of biological processes were investigated, with inflammatory (*n* = 49), neurodegenerative (*n* = 34), apoptotic (*n* = 31), and oxidative stress-related biomarkers (*n* = 21) being the most frequently reported, followed by gliosis (astrocytic response) (*n* = 19) and microglial activation (*n* = 13). These biomarkers were assessed across broad temporal windows, typically ranging from 30 min to 30 days post-MCAO, depending on the biological process. Inflammatory responses were commonly characterized by increased expression of cytokines such as interleukin (IL)-1β, IL-6, TNF-α, and NF-κB [21,32,79,85,86,107,123,126,132]. In parallel, activation of inflammasome-related pathways, including NLRP3, ASC, caspase-1, and Gasdermin D, as well as reduced anti-inflammatory mediators such as IL-10 and transforming growth factor-β (TGF-β), was reported [51,74,94,99,113,132,135].

Oxidative stress markers, including MDA, 4-HNE, and 8-OHdG, were frequently elevated, alongside reductions in antioxidant defenses such as GSH, SOD, CAT, GSH-Px, and T-AOC [27,46,51,90,92,93,109,115,134].

Neurodegenerative and structural alterations included reductions in neuronal and synaptic markers such as MAP2, MBP, and synaptic proteins, as well as changes in neurogenesis-related markers (e.g., DCX, BrdU/NeuN) and neurotrophic factors, including BDNF, GDNF, and VEGF-A [49,62,83,93,96,99,127]. Gliosis and microglial activation were also frequently reported, with increased expression of GFAP and microglial markers such as Iba1 and CD68 [38,62,74,99,120,131].

Apoptotic pathways were characterized by increased caspase-3 activation, Bax/Bcl-2 imbalance, and cytochrome c release [37,54,60,83,115,138], while mitochondrial dysfunction (*n* = 7) and autophagy-related markers (*n* = 6), including LC3-II, Beclin-1, and LAMP1, indicated alterations in cellular energy regulation and degradation pathways [25,66,71,112,115,130].

Additional molecular processes included BBB-related markers (*n* = 11), angiogenesis (*n* = 8), calcium signaling (*n* = 5), neurotransmitter alterations (*n* = 5), neuroplasticity (*n* = 4), myelin-related processes (*n* = 3), coagulation (*n* = 2), synaptic function (*n* = 2), and excitotoxicity (*n* = 1), reflecting the multifactorial nature of ischemic injury.

Peripheral biomarkers assessed in blood samples included, among others, inflammatory mediators (*n* = 11), organ function markers (kidney: *n* = 3; liver: *n* = 2), angiogenesis (*n* = 2), neuroendocrine responses related to the hypothalamic–pituitary–adrenal axis (*n* = 2), metabolic parameters (*n* = 2), oxidative stress markers (*n* = 1), and glutamatergic activity (*n* = 1), typically evaluated between 24 h and 35 days post-MCAO.

Additional analyses were conducted in other biological compartments, including gut microbiota (cecum/colon; *n* = 2), urine/kidney samples (*n* = 1), and retinal tissue (*n* = 1), further highlighting the systemic impact of transient cerebral ischemia. Overall, considerable heterogeneity was observed in both the type of biomarkers assessed and the timing of evaluation across studies.

### 3.10. Neurobehavioral, Cognitive, and Physiological Outcomes

Functional outcomes are summarized in Table 8. Neurological deficit scores were the most frequently reported outcome (*n* = 90), including commonly used scales such as the modified neurological severity score (mNSS), Bederson, Garcia, and Longa scores, assessed over a wide temporal range from 2 h to 42 days post-MCAO. Across studies, these scores consistently indicated moderate-severe neurological impairment during the acute phase (24–72 h), followed by partial and variable recovery over time. Representative values illustrate the severity and temporal evolution of neurological deficits following MCAO. For example, mNSS scores (0–18-point scale) reached 13.0 ± 1.6 at 1-day post-MCAO, decreasing to 9.2 ± 1.3 at 7 days and 6.3 ± 0.8 at 14 days, indicating partial functional recovery over time [116]. Similarly, Longa scores (5-point scale) of approximately 3.6 ± 0.7 at 24 h decreased to 2.6 ± 0.7 at 28 days post-ischemia, reflecting improvement in neurological function during the subacute phase [37]. In both cases, higher scores reflect greater neurological impairment.

When neurobehavioral data were considered alongside lesion severity and occlusion duration, the available studies generally indicated that larger infarcts and longer occlusions were accompanied by more severe functional impairment, although direct quantitative comparison across studies remained limited. In one study, a severe infarction phenotype involving 27–48% of the injured hemisphere was associated with a 24 h mNSS of 14.0 ± 1.4, with individual scores ranging from 12 to 17 [19]. In another severe ischemia model, total infarct volume reached 302 ± 61 mm^3^; however, neurological impairment was assessed using a 48-point neuroscore rather than the 18-point mNSS, with a reported score of 14 ± 5 at 7 days post-stroke, limiting direct comparability [77]. Regarding ischemia duration, untreated groups subjected to 120 min occlusion showed moderate to severe 24 h Longa scores, including 2.05 ± 0.55, 2.25 ± 0.5, and 3.6 ± 0.7 [37,48,131], while rotarod performance remained significantly impaired during the subacute phase [31]. In 60 min occlusion protocols, neurological deficits were also evident during the first days after ischemia, although some studies reported partial or complete recovery by 14 days, with motor coordination deficits detectable on rotarod testing at 24 h [65,85]. Across these studies, higher neurological deficit scores were reported in experimental conditions associated with larger infarcts or longer occlusion durations; however, scoring systems, assessment time points, and reporting formats differed substantially between studies [19,55].

Motor function was assessed in a substantial number of studies (*n* = 32), using tests such as rotarod, beam walking, grip strength, foot-fault, and cylinder tests. These assessments consistently revealed impairments in coordination, balance, and forelimb use shortly after occlusion, often persisting up to 7–21 days post-MCAO. For example, rotarod performance was markedly reduced at 24 h post-MCAO (35.4 ± 36.7 s), with partial recovery observed at 7 and 28 days (53.8 ± 28 s), although values remained below baseline levels [37]. Similarly, foot-fault rates remained elevated during the first week post-ischemia, decreasing from approximately 33% at 1 day to ~12.5% at 7 days, indicating persistent but improving motor deficits [75]. Sensorimotor integration deficits were also reported using adhesive removal and vibrissae-evoked forelimb placing tests, demonstrating prolonged response times and lateralized motor deficits [22,31,62].

Cognitive function, primarily evaluated through spatial learning and memory paradigms (*n* = 8), was assessed using the Morris water maze and Barnes maze. These studies consistently reported impaired acquisition and retention, reflected by increased escape latency and reduced task performance, with deficits persisting into subacute and chronic phases (24 h–42 days post-MCAO). For example, in the Barnes maze test, escape latency increased to approximately 23.6 s at 4 days post-MCAO compared to 13.0 s in control animals, indicating impaired spatial learning [103]. Similarly, long-term impairments in spatial learning and memory were reported up to 42 days post-MCAO in Morris water maze assessments [27].

Affective behavior was less frequently investigated (*n* = 2), with anxiety- and depression-like behaviors assessed using the elevated plus maze, forced swimming test, and sucrose preference test. Included studies reported increased anxiety-like behavior and depressive-like phenotypes following ischemic injury.

Physiological parameters, particularly body weight changes, were reported in a subset of studies (*n* = 10). Body weight loss was commonly observed during the early post-ischemic period (1–7 days), followed by gradual recovery in later phases. In some cases, weight reductions reached approximately 25–30% within the first days post-MCAO, indicating substantial systemic impact [22,80,85,124].

Overall, considerable heterogeneity was observed across studies in both the type of functional assessments used, the timing of evaluation, and the scoring systems applied, limiting direct comparability of neurobehavioral outcomes.

### 3.11. Additional Methodological and Outcome Reporting Aspects

In addition to the outcomes summarized in the preceding tables, additional methodological and outcome-related variables were extracted to provide a more comprehensive characterization of the included studies, as these factors may influence the reproducibility and interpretation of experimental stroke models.

Ischemia monitoring. Ischemia monitoring across the included studies was predominantly performed using laser Doppler flowmetry (LDF), reflecting its widespread use in preclinical stroke models. Despite this, variability in monitoring approaches was observed, including differences in systems and probe configurations. Detailed information on the monitoring techniques used is provided in the Supplementary Materials (Table S3).

Temperature control. Temperature control during experimental procedures was frequently reported, with rectal temperature typically maintained around 37 °C to ensure physiological homeostasis. Several studies defined narrow target ranges (e.g., 36.5–37.5 °C [22,124], 37–38 °C [114], or 35–37 °C [48]). Temperature regulation was generally achieved using heating pads or feedback-controlled systems [29]. However, temperature monitoring was not uniform across studies, and only one study simultaneously reported rectal and brain temperatures (37 ± 0.5 °C and 36 ± 0.5 °C, respectively) [90].

Pain management. Pain management strategies varied considerably across studies and were inconsistently reported. When described, perioperative analgesia included local anesthetics (e.g., bupivacaine and lidocaine) [45,114,136], as well as systemic analgesics such as buprenorphine [24,38,118], meloxicam [59], and carprofen [55]. Additional agents, including ropivacaine [22] and tolfenamic acid [62], were used in specific protocols. In several cases, anesthetic agents such as pentobarbital were also employed for deep anesthesia or euthanasia [74,121,136]. Overall, substantial heterogeneity was observed in both the type and reporting of analgesic protocols.

Mortality rate. Mortality rates were reported in a subset of studies and varied widely across experimental conditions. Reported values ranged from approximately 4% [44] to 50% [39], reflecting differences in ischemia duration, surgical techniques, animal characteristics, and experimental design. In long-term survival studies, mortality rates reaching up to 65% were also reported [86]. Higher mortality rates (>30%) were mainly described in studies reporting severe ischemic or procedure-related complications. Acute deaths were frequently attributed to severe brain edema and cerebral herniation within the first 24–48 h after MCAO [38,44]. Procedure-related hemorrhagic complications, particularly subarachnoid hemorrhage and subdural hematoma, were also reported as important causes of mortality and were associated with excessive filament insertion depth, vessel rupture, or surgical approach [45,141]. In one study, the external carotid artery insertion approach was associated with 30% mortality due to subarachnoid hemorrhage, compared with 10% hemorrhage-related mortality using the common carotid artery insertion approach [141]. Additional factors associated with increased mortality included poor collateral reperfusion after common carotid artery ligation, particularly in animals dependent on the circle of Willis for residual perfusion [59], and post-reperfusion respiratory distress related to anesthesia recovery in the early postoperative period [44]. Mortality also differed according to animal-related and experimental variables. One study reported a mortality higher than expected in females compared with age-matched males and used different occlusion times to improve the overall survival of the animals [39]. In some studies, mortality rates differed substantially between experimental groups, depending on factors such as monitoring method, occlusion duration, or follow-up period [85,86,129]. Overall, the mortality reported was heterogeneous and not consistently included across studies.

### 3.12. Risk of Bias Assessment

**Figure 2:**
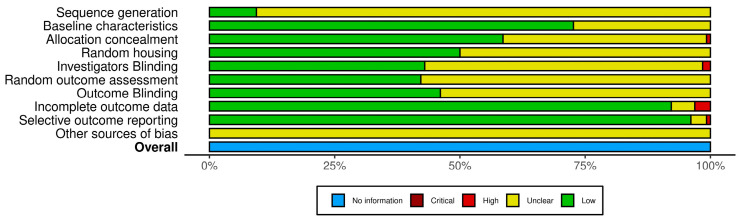
SYRCLE’s risk of bias assessment of the included studies using the SYRCLE tool.

The risk of bias was assessed for all articles included in this systematic review using the SYRCLE tool [14]. The articles were evaluated by domain using a color-coded system, where green indicates low risk of bias, yellow indicates unclear risk of bias, and red indicates high risk of bias (Figure 2). The risk of bias assessment for each study is available in the Supplementary Materials (Figure S1).

### 3.13. Reporting Quality Assessment

The studies included in this systematic review were assessed for quality using the ARRIVE guidelines [16]. Each domain was evaluated and a scoring scale was applied (yes = 2 points, unclear = 1 point, no = 0 points), with total scores ranging from 19 to 42. Studies were classified as high quality (29–42 points), unclear quality (15–28 points), or low quality (0–14 points). Most studies were classified as high quality, although variability in reporting across specific ARRIVE domains was observed (Table 9). This assessment provided a structured evaluation of the overall reporting quality of the included studies.

### 3.14. Methodological Reliability

Inter-rater agreement was assessed using Cohen’s kappa coefficient for study screening (Rayyan), risk of bias assessment (SYRCLE), and reporting quality evaluation (ARRIVE), yielding values of 0.76, 0.89, and 0.90, respectively (Supplementary Materials Table S4).

## 4. Discussion

This systematic review provides a comprehensive synthesis of current practices in the tifMCAO model in adult rats, identifying both recurring methodological patterns and substantial variability across studies. A commonly used experimental framework was observed, typically involving male Sprague–Dawley rats aged 6–12 weeks, silicone-coated nylon monofilaments (4–0, 0.35–0.40 mm tip diameter), occlusion durations of 60–120 min—most frequently 90 min—and isoflurane anesthesia. These features reflect widely adopted experimental practices associated with reliable infarct induction and acceptable survival rates [9,143,144].

However, this apparent convergence does not represent true standardization. Substantial variability persists across key experimental domains, including animal characteristics, ischemia induction parameters, anesthesia protocols, and perioperative management. These factors are known to directly influence infarct size, pathophysiological responses, and functional outcomes, thereby affecting the consistency and interpretation of findings [143,144,145].

Outcome assessment exhibited a similar pattern, with structural, vascular, molecular, and functional endpoints differing in measurement techniques, reporting formats, and timing. This lack of harmonization limits cross-study comparability and complicates the integration of evidence, a challenge widely recognized in preclinical stroke research [144,145].

Taken together, these findings directly address the central question of this review: although it is possible to identify commonly used methodological approaches, the tifMCAO model is characterized by substantial variability in both implementation and outcomes evaluation. This combination of partial convergence and persistent variability reflects the flexibility of the model but also represents a major barrier to reproducibility and translation, underscoring the need for improved methodological alignment and adherence to established recommendations such as STAIR and ARRIVE [16,143].

### 4.1. Methodological Heterogeneity and Its Implications

A major finding of this systematic review is the substantial methodological heterogeneity across tifMCAO studies, spanning animal-related characteristics, ischemia induction protocols, anesthetic regimens, and perioperative management. Importantly, these sources of variability do not act independently but interact to shape infarct development, functional outcomes, and the overall interpretation of experimental results, thereby limiting reproducibility and comparability across studies.

Variability in animal-related parameters represents a primary contributor to this heterogeneity. The predominance of Sprague–Dawley and Wistar rats reflects their practical advantages; however, strain-dependent differences in cerebrovascular anatomy and ischemic susceptibility may influence infarct size and recovery profiles. For example, anatomical variation in the middle cerebral artery and collateral circulation has been described in Sprague–Dawley rats, affecting occlusion efficiency and infarct distribution [146], while differences in lesion volume and edema formation between Sprague–Dawley and Wistar rats have been demonstrated under comparable experimental conditions [147]. Similarly, strain- and vendor-dependent variability in MCAO outcomes has been reported [148]. The marked preference for male animals reduces hormonal variability but limits generalizability, as female sex hormones, particularly estrogen, have been shown to confer neuroprotection and modulate post-ischemic inflammation [149,150]. Age was frequently restricted to young adult animals (6–12 weeks), a developmental window that does not fully reflect physiological maturity or the comorbidity burden observed in clinical populations. These choices, while methodologically convenient, may reflect a tendency to prioritize experimental consistency over biological representativeness.

This pattern extends to ischemia induction parameters. Although the most common occlusion duration was 60 to 120 min, a wide variety of protocols were identified, including studies that did not report the duration. Given the strong relationship between ischemia duration and infarct severity, such inconsistencies directly affect lesion size and downstream outcomes [8,143,151]. Likewise, despite the widespread use of silicone-coated nylon filaments, variations in diameter, coating, and insertion depth introduce additional variability in the vascular occlusion. Notably, these procedural differences are often tailored to specific experimental conditions, suggesting that variability may partly reflect deliberate optimization rather than methodological inconsistency, and highlighting that variability may also represent adaptation to specific experimental objectives.

Anesthetic and perioperative factors further complicate interpretation. Isoflurane was the predominant anesthetic, although alternative regimens were also employed. Importantly, isoflurane has well-documented neuroprotective properties, including vasodilatory effects on cerebral circulation and modulation of excitotoxic and inflammatory pathways, which can reduce infarct size independently of experimental interventions [152]. Such effects may confound the interpretation of neuroprotective strategies when not adequately controlled. Inconsistent reporting of anesthesia and analgesia protocols further limits cross-study comparisons, despite their recognized impact on physiological stress and recovery. This gap underscores a persistent disconnect between methodological reporting standards and their implementation in practice [16].

Additional sources of variability arise from ischemia monitoring and physiological control. Laser Doppler flowmetry was widely used; however, differences in implementation and reporting restrict comparability. Temperature regulation, a critical determinant of ischemic outcome, was not consistently reported, despite strong evidence that even minor deviations from normothermia significantly alter infarct size and neurological deficits [144,153,154,155,156]. Together, these observations indicate that incomplete control and reporting of key physiological variables remain important contributors to experimental variability.

Collectively, this variability reflects not only methodological diversity but also structural constraints within preclinical research. The predominance of young, healthy, and male animals likely reflects economic and logistical considerations, as aged or comorbid models are more costly and associated with higher variability and mortality. In parallel, methodological inertia—where laboratories maintain long-established protocols—supports internal consistency but may hinder adaptation to evolving guidelines. The technical complexity of the MCAO model further amplifies variability, as surgical skill and procedural nuances significantly influence outcomes. Finally, the pressure to obtain statistically robust results may favor simplified and homogeneous models, reducing variability at the expense of clinical relevance, a limitation that has been widely recognized in preclinical research [157].

Taken together, these factors illustrate that methodological heterogeneity in tifMCAO studies is not merely a limitation but a consequence of competing experimental priorities, whose cumulative effect complicates the interpretation of preclinical evidence and ultimately challenges the translation of experimental findings to clinical settings. This gap between experimental practice and translational expectations has prompted recent collaborative initiatives, such as the Stroke Preclinical Assessment Network (SPAN), which aim to improve rigor, reproducibility, and predictive validity in preclinical stroke research [158], alongside established frameworks such as STAIR [143].

### 4.2. Outcome Assessment and Methodological Considerations

Outcome assessment in tifMCAO models encompasses a broad range of structural, molecular, and functional endpoints, reflecting the multifactorial nature of ischemic brain injury. However, marked variability in outcome selection, measurement techniques, reporting formats, and assessment timing represents a major limitation for cross-study comparability and translational interpretation. Importantly, these outcomes should not be considered in isolation, as infarct size, edema formation, BBB disruption, molecular responses, and functional deficits are tightly interconnected processes that evolve dynamically over time [143,144,159].

Infarct quantification is a key indicator of stroke severity and correlates closely with neurological deficits, making it a critical component of preclinical studies to evaluate therapeutic efficacy [160,161,162]. As summarized in Table 4, infarct size is predominantly assessed using TTC staining, histological methods, or MRI. TTC staining is widely used due to its simplicity and rapid assessment of metabolic activity in acute phases (24–72 h). However, its reliability decreases in chronic stages due to macrophage infiltration and tissue remodeling, which may obscure viable versus non-viable tissue [163,164]. Histological methods, including H&E and Nissl staining, provide higher cellular resolution and allow detailed characterization of necrosis, inflammation, and neuronal loss [165], but are inherently terminal and subject to variability in staining protocols and interpretation. MRI-based approaches offer clear advantages by enabling in vivo and longitudinal assessment, including differentiation of ischemic core and penumbra through diffusion- and perfusion-weighted imaging [139,166,167]. Nevertheless, their limited use and dependence on protocol-specific parameters restrict comparability across studies, despite recommendations for multimodal imaging in preclinical stroke research [143,144].

A critical but often underappreciated methodological issue in infarct assessment is the confounding effect of edema. Infarct volume may be substantially overestimated in the absence of edema correction, particularly during the acute phase [168]. The use of indirect correction methods, such as the Swanson formula, partially addresses this bias; however, not all studies apply or report such corrections, introducing systematic variability. This is particularly relevant given that edema formation is not merely a confounding factor but an integral component of ischemic pathophysiology, directly influencing tissue displacement and lesion expansion [169]. Therefore, infarct size should be interpreted in conjunction with edema metrics rather than as an isolated endpoint.

The assessment of edema itself reflects a similar methodological dichotomy. The wet/dry method remains the most used approach (Table 5), providing a quantitative estimate of total brain water content. While robust and straightforward, this method lacks spatial resolution and does not distinguish between cytotoxic and vasogenic edema. In contrast, MRI-based techniques, including T2-weighted imaging and ADC mapping, allow spatial and temporal characterization of edema progression [166]. As observed, early reductions in diffusion reflect cytotoxic edema, whereas later increases in diffusion combined with T2 hyperintensity indicate vasogenic edema and BBB breakdown [139]. Despite these advantages, MRI-based edema assessment remains underutilized, and variability in acquisition protocols further limits standardization.

BBB disruption is another key outcome domain, yet its assessment is frequently simplified. In experimental models, ischemia induces disruption of tight junctions, leading to increased BBB permeability and aggravated injury severity [170]. In contrast, human data suggest that BBB dysfunction occurs early after stroke onset and follows a dynamic, often biphasic or protracted course, with substantial variability across patients [159]. These findings indicate that the temporal dynamics of BBB dysfunction are only partially captured in preclinical models.

BBB dysfunction is therefore a dynamic and multifaceted process involving alterations in permeability, structural integrity, and transport kinetics. As shown in Table 6, Evans Blue extravasation is the most used method, providing an indirect measure of albumin leakage across the BBB. However, this approach reflects only macromolecular permeability and is highly dependent on experimental conditions. The use of fluorescent tracers (e.g., NaFL, dextran) enables a more refined assessment but introduces variability related to tracer size and molecular weight [48,76]. Molecular analyses of tight junction proteins (claudin-5, occludin, ZO-1) provide insight into structural disruption and are associated with increased permeability [170]. In addition, MMP-mediated degradation of these proteins contributes to edema, hemorrhage, and secondary injury [171,172,173]. Only a limited number of studies assess BBB transport kinetics using in situ perfusion [100], despite its physiological relevance. Furthermore, most studies evaluate BBB disruption at a single time point within the acute phase (6–48 h), overlooking its biphasic pattern characterized by early and delayed opening phases [170,174,175].

Molecular and peripheral biomarkers provide important mechanistic insights into ischemic injury (Table 7). Acute-phase assessments predominantly capture oxidative stress, inflammasome activation, and pro-inflammatory signaling, whereas later phases reflect apoptosis, gliosis, angiogenesis, and neuroplasticity. Markers of oxidative stress, including MDA, 4-HNE, and 8-OHdG, were consistently elevated, while antioxidant defenses such as GSH, SOD, CAT, and GSH-Px were reduced. In parallel, pro-inflammatory cytokines (IL-1β, IL-6, TNF-α) and activation of transcriptional regulators such as NF-κB were frequently reported, reflecting early neuroinflammatory responses that contribute to secondary injury [74,79,99,123,144,176]. Importantly, these acute molecular changes are closely linked to BBB disruption and edema formation [170,176]. In later phases, biomarkers related to apoptosis, astrocytic and microglial activation (GFAP, Iba-1), angiogenesis (VEGF), and synaptic plasticity (BDNF) were more frequently reported, reflecting the transition from injury to repair mechanisms [20,32,113,144]. Despite this mechanistic richness, several methodological limitations remain. Biomarker selection is often heterogeneous and hypothesis-driven, limiting cross-study comparability. Most studies rely on single-time-point measurements, typically in the acute phase, failing to capture the temporal evolution of molecular responses. In addition, the predominant focus on brain tissue contrasts with the relatively limited use of peripheral biomarkers, despite their recognized translational relevance in clinical stroke assessment [177,178].

Functional outcomes are essential for assessing translational relevance. Neurological deficit scores (mNSS, Bederson, Longa) are widely used and sensitive to acute impairment [48,63,90], but provide relatively coarse measurements and may fail to detect subtle improvements [179,180]. In contrast, motor and sensorimotor tests (rotarod, foot-fault, cylinder) and cognitive paradigms (Morris water maze, Barnes maze) offer more refined assessments of functional recovery, particularly in subacute and chronic phases [37,75,103,143,179]. Cognitive and affective outcomes, although less frequently assessed, are increasingly recognized as clinically relevant domains [144,181].

Physiological and survival outcomes, including body weight changes and mortality rates, provide important contextual information. Body weight loss reflects systemic stress, neuroinflammation, and disease severity, while mortality rates vary depending on ischemia severity and experimental conditions [144,182]. However, these outcomes are inconsistently reported and often treated as secondary variables, which may introduce survivorship bias and lead to underestimation of model severity and treatment effects [144,183].

A key limitation across outcome domains is the timing of assessment. Most studies focus on a single time point, typically around 24 h post-MCAO, corresponding to peak infarct size and edema. While informative for acute injury, this approach fails to capture the dynamic evolution of ischemic damage and recovery. Early neuroprotective effects may not translate into sustained functional benefit, highlighting the importance of long-term follow-up [143,158]. A more integrated and longitudinal evaluation of structural, molecular, and functional endpoints is therefore critical to improve interpretability and translational relevance [144].

### 4.3. Translational Relevance

The translational relevance of the tifMCAO model lies in its ability to reproduce key features of ischemic stroke, particularly focal ischemia followed by reperfusion. This is especially relevant in the current clinical context, where reperfusion therapies such as thrombolysis and mechanical thrombectomy are the standard of care. The model reliably induces infarction, edema, blood–brain barrier (BBB) disruption, and neurological deficits, supporting its value for mechanistic and therapeutic studies [9,144].

However, important limitations constrain clinical translatability. The model relies on mechanical occlusion using a filament, which does not replicate the biological complexity of thromboembolic stroke. In humans, thrombi interact dynamically with the endothelium and undergo spontaneous or treatment-induced lysis, while reperfusion is often incomplete or delayed. In contrast, filament withdrawal produces abrupt reperfusion, which may overestimate the treatment efficacy.

A major translation gap arises from the characteristics of experimental subjects. The underrepresentation of female animals neglects sex-dependent differences in stroke pathophysiology. Similarly, most preclinical studies use young, healthy animals, which poorly reflect the clinical population, typically composed of older patients with multiple comorbidities that influence BBB integrity, inflammation, and recovery [9,153]. The use of homogeneous animal populations reduces variability and cost, facilitating statistically significant results, but may contribute to inflated efficacy estimates and publication bias [145]. Together, these factors highlight a fundamental mismatch between experimental models and clinical reality, representing a key barrier to the translation of neuroprotective strategies [153].

Outcome selection further highlights this disconnect. While infarct size is the predominant endpoint in preclinical studies, clinical success is defined by functional recovery. Although neurological and motor assessments are widely used, they often rely on relatively coarse scales, while cognitive and long-term outcomes remain underexplored [143,161,184]. Consequently, reductions in infarct size do not necessarily translate into meaningful functional improvement.

Finally, differences in treatment timing and follow-up duration limit comparability with clinical practice. Preclinical interventions are often administered immediately after ischemia, whereas patients experience delays in treatment. In addition, outcomes are frequently assessed at acute time points, failing to capture long-term recovery dynamics [143,158]. Accordingly, methodological variability in tifMCAO studies should be considered when defining eligibility criteria, therapeutic windows, outcome hierarchies, and follow-up duration in future clinical trials, to ensure that preclinical efficacy signals are aligned with clinically meaningful endpoints. Overall, while the tifMCAO model provides robust experimental control and mechanistic insight, its translational value is constrained by simplified biological context, limited representation of patient populations, and incomplete alignment with clinically relevant outcomes.

### 4.4. Standardization Recommendations

The findings of this review highlight the need for targeted standardization strategies tailored to the tifMCAO model. While variability reflects experimental flexibility, certain parameters can be harmonized without compromising biological relevance.

First, the duration of ischemia should be selected depending on the experimental objective. Shorter occlusions (60 min) are more appropriate for studying neuroprotection and recovery from the ischemic penumbra, while longer durations (90–120 min) produce more consistent infarcts and are suitable for mechanistic or severe injury models. Clear justification of ischemia time is therefore essential, as recommended by STAIR guidelines [143].

Second, filament selection should be standardized relative to animal body weight. The predominance of silicone-coated monofilaments (0.35–0.40 mm) supports their reliability; however, consistent reporting of diameter, coating, and insertion depth is critical to reduce variability in vascular occlusion and infarct size.

Third, anesthetic choice requires careful consideration due to its physiological effects. Isoflurane, although widely used, induces cerebral vasodilation and may exert neuroprotective effects, potentially confounding outcomes. Standardizing anesthetic regimens and explicitly reporting protocols is therefore necessary to improve comparability across studies.

From an animal welfare perspective, high-risk procedural factors should be proactively minimized through precise filament placement, real-time ischemia monitoring, rigorous perioperative surveillance, and strict thermoregulation. Excessive filament advancement may increase the risk of vessel rupture and hemorrhagic complications, whereas inadequate monitoring of respiratory, hemodynamic, and temperature parameters during anesthesia and recovery may contribute to early postoperative mortality [44,45,70,141]. In addition, strain-related vascular anatomy and collateral circulation should be considered when selecting experimental conditions, as these factors may influence ischemic severity, edema formation, and survival [146].

Although recurring methodological patterns were identified, the available evidence does not support a definitive protocol-level recommendation for achieving predefined infarct severity categories, because infarct volume is jointly influenced by ischemia duration, filament characteristics, animal strain, anesthesia, physiological control, and outcome timing. Future studies should therefore report these parameters in sufficient detail to enable robust protocol-level comparisons and evidence-based selection of experimental conditions for specific translational objectives.

Beyond technical parameters, adherence to minimum reporting standards remains a priority. The ARRIVE 2.0 guidelines define essential elements—including study design, randomization, blinding, and sample size calculation—that are still inconsistently reported in preclinical stroke research [16]. Similarly, STAIR recommendations emphasize rigorous experimental design, inclusion of relevant biological variables, and multimodal outcome assessment to enhance translational validity [143].

Finally, a critical step forward is the adoption of multicenter preclinical approaches. Initiatives such as the Stroke Preclinical Assessment Network (SPAN) demonstrate the feasibility of harmonized protocols across laboratories, incorporating randomization, blinding, and standardized outcome measures to reduce bias and improve reproducibility [158]. Complementary efforts, including MULTI-PART and PRISM, further support collaborative frameworks as a strategy to overcome the limitations of single-center studies [158,184,185,186].

From a practical experimental design perspective, the choice of tifMCAO conditions should be aligned with the intended study objective. Short-term neuroprotection studies may benefit from protocols producing reproducible moderate lesions, whereas long-term recovery studies should balance infarct severity against survival, functional recovery, and animal welfare. Based on the patterns identified in this review, key parameters requiring strict control include occlusion duration, filament diameter and insertion depth relative to animal size, anesthetic regimen, temperature regulation, ischemia monitoring, and perioperative recovery. High-risk conditions, including excessive filament advancement, inadequate physiological monitoring, poor temperature control, prolonged or severe ischemia, and insufficient anesthesia recovery surveillance, should be avoided or explicitly justified. These considerations may serve as a practical evidence-informed checklist for improving reproducibility, interpretability, and animal welfare in future tifMCAO studies.

Collectively, these recommendations suggest that improving translational success in stroke research will require not only protocol refinement but also a shift toward coordinated, rigorous, and transparently reported preclinical investigation.

### 4.5. Limitations of the Systematic Review and Future Directions

Several limitations should be considered when interpreting the findings of this systematic review. First, substantial methodological variability across studies—spanning ischemia induction, outcome assessment, and timing—limited cross-study comparability and precluded quantitative synthesis, including meta-analysis. In addition, incomplete reporting of key experimental parameters, such as filament characteristics, anesthesia protocols, and edema correction methods, further reduced comparability and may have introduced reporting bias. Although most studies were classified as high reporting quality according to ARRIVE, the SYRCLE assessment revealed that critical domains, including randomization, allocation concealment, and blinding, were frequently unclear, limiting confidence in internal validity and reproducibility.

The exclusion of animals with comorbidities was a deliberate methodological decision aimed at isolating the impact of specific technical variables from the tifMCAO model, strengthening internal validity and allowing for more consistent comparisons between studies. However, this approach limits external validity, as it does not fully reflect the clinical population, in which age, sex, and comorbidities substantially influence stroke pathophysiology and recovery [145,153]. This trade-off between experimental control and clinical relevance represents an inherent challenge in preclinical stroke research. Potential publication bias and selective outcome reporting must also be considered, as studies reporting positive or statistically significant findings are more likely to be published, potentially inflating perceived efficacy [145].

An additional consideration relates to the temporal scope of this review, which was restricted to studies published from 2018 onwards. This criterion was primarily defined to build upon and update previous literature syntheses in this field. Notably, this period coincides with the increasing adoption of reporting guidelines such as ARRIVE 2.0, which may have contributed to improved reporting quality and methodological transparency. However, this does not necessarily translate into reduced methodological heterogeneity, which remained substantial across studies. While this approach enhances internal consistency, it may limit comparability with earlier literature and obscure longer-term trends in the evolution of the tifMCAO model.

Future research should prioritize three key areas. First, improving standardization and transparency through rigorous implementation of ARRIVE, SYRCLE, and STAIR recommendations, particularly regarding randomization, blinding, and detailed methodological reporting. Second, enhancing translational relevance by incorporating biologically representative models, including aged animals, females, and comorbidity models. Third, promoting harmonized outcome assessment through multimodal approaches (e.g., imaging, molecular, and functional endpoints) and longitudinal designs that capture both acute and chronic phases of injury. Finally, collaborative multicenter preclinical studies, as proposed by recent initiatives, represent a crucial step towards validating results in different laboratories and improving reproducibility, ultimately strengthening the bridge between experimental research and clinical application.

## 5. Conclusions

This systematic review provides a comprehensive synthesis of methodological practices and outcome assessment strategies in the transient intraluminal filament middle cerebral artery occlusion (tifMCAO) model in rats. Although it is possible to identify a set of commonly used experimental parameters—such as the silicone-coated monofilaments, occlusion durations of 60 to 120 min, and isoflurane anesthesia—these do not constitute true standardization. Instead, the model is characterized by a combination of implicit methodological convergence and substantial variability across studies.

Importantly, this review demonstrates that methodological choices are not neutral but directly influence infarct development, blood–brain barrier disruption, molecular responses, and functional outcomes, and shows how variations in experimental design contribute to differences in reported outcomes. Variability in key parameters—including ischemia duration, filament properties, anesthetic regimens, and outcome assessment—limits comparability across studies and contributes to the persistent challenges in reproducibility and translation.

In addition to documenting heterogeneity, this work provides an integrated framework that links experimental design to the interpretation of results, highlighting how structural, molecular, and functional parameters should be considered as interconnected components of ischemic pathophysiology. At the same time, the predominance of simplified experimental conditions underscores a fundamental mismatch between preclinical models and the clinical reality of stroke.

Collectively, these findings reinforce that the translational value of the tifMCAO model depends not only on its technical implementation but on the alignment between experimental design, outcome selection, and clinical relevance. By providing an organized framework for interpreting methodological variability, this review contributes to improving the reproducibility, comparability, and translational impact of preclinical stroke research.

## Figures and Tables

**Table 1:** Ischemia duration in rat tifMCAO models: Protocols and frequency of use.

Ischemia Duration (min)	Frequency (Qualitative) *	Ref.
30	Low	[40,94,123]
60	High	[18,28,29,35,38,44,47,54,60,61,62,69,82,83,84,85,86,88,89,101,114,124,130,139,140]
75	Low	[24,36,87,103]
90	Very high	[20,21,22,32,41,42,43,45,46,50,52,53,59,63,65,66,71,77,79,80,81,90,91,92,93,100,104,105,107,108,110,111,115,117,118,127,128,129,132,134,137,141]
100	Low	[64]
120	Very high	[19,23,25,26,27,30,33,37,48,51,56,57,58,67,68,70,73,74,75,76,78,96,98,99,102,109,112,113,116,119,120,121,122,125,126,131,133,135,136,142]
≥180	Low	[31,95]
Variable	Low	[34,39,55,138]
ND	Low	[49,72,97,106]

Notes: Ischemia durations are presented according to their frequency of use across studies. Protocols with multiple durations (e.g., 60, 120, and 180 min) were grouped as variable. * Very high (*n* ≥ 30), high (*n* = 15–29), moderate (*n* = 5–14), and low (*n* < 5). Abbreviations: min, minutes; ND, not defined.

**Table 2:** Filament characteristics in rat tifMCAO models.

Filament Base Material	Coating/Tip Modification	Tip Diameter (mm)	Suture Size	Frequency (Qualitative) *	Ref.
Nylon monofilament	Silicone-coated	0.35–0.40	3-0 to 5-0	Very high	[18,21,22,23,24,27,28,29,32,33,34,35,36,38,39,40,43,45,46,50,53,55,57,58,62,63,65,70,75,77,79,80,81,82,87,88,89,95,100,101,103,104,105,107,113,116,117,118,132,137]
	None (rounded/cylindrical)	ND	3-0 to 5-0	Moderate	[20,26,42,47,54,56,71,74,85,86,110,124,125,133]
	Poly-L-lysine-coated	ND	3-0 to 4-0	Moderate	[37,60,61,66,91,92,129,134]
	Heparin-coated	0.25–0.28	ND	Low	[51]
	Poly-L-lysine + Heparin-coated	ND	4-0	Low	[31]
	Paraffin-coated	0.26–0.28	ND	Low	[76]
	Polysiloxane	ND	5-0	Low	[30]
	Fire-polished tip	0.38–0.40	4-0	Low	[140]
Silicon–Teflon	Poly-L-lysine	0.30	5-0	Low	[108]
ND	Silicone-coated	0.36–0.37	3-0 to 4-0	Moderate	[59,83,90,93,98,109,114,119,122,137,141]
	ND	ND	ND	Moderate	[52,64,67,72,94,115,127,131,135,139,142]

Notes: Filament characteristics are summarized according to base material and surface modification. Coating and tip modification include procedures such as silicone coating, poly-L-lysine coating, heparin coating, paraffin coating, or tip shaping (e.g., rounded, blunted, cylindrical, or fire-polished). Values for tip diameter are reported as described in the original studies. * Very high (*n* ≥ 30), high (*n* = 15–29), moderate (*n* = 5–14), and low (*n* < 5). Abbreviations: mm, millimeters; ND, not defined.

**Table 3:** Anesthesia protocols in rat tifMCAO models.

Anesthetic Agent	Route of Administration	Typical Dose/Concentration	Frequency (Qualitative) *	Ref.
Isoflurane	Inhalation	Induction: 3–5%; Maintenance: 1–3%	Very high	[19,21,22,29,31,32,33,34,38,39,41,44,45,48,55,62,63,64,65,70,77,80,81,82,84,85,86,88,89,90,93,96,100,101,106,107,110,111,112,114,116,118,121,122,123,125,132,134,138,139,140,141]
Chloral hydrate	IP	300–400 mg/kg	High	[18,25,51,54,57,66,72,76,83,97,98,109,115,120,124,129,133]
Ketamine + Xylazine	IP/IM/IV	Ketamine: 40–80 mg/kg; Xylazine: 5–20 mg/kg	Moderate	[40,46,59,60,61,75,79,91,92,103,108,128]
Pentobarbital	IP/IV	30–50 mg/kg	Moderate	[26,56,71,74,78,102,105,119,126,131,135,136]
Halothane	Inhalation	1–3.5%	Moderate	[42,50,52,53,104]
Sevoflurane	Inhalation	Induction: 5–6%; Maintenance: 3–4%	Low	[24,36,87,113]
Ketamine	IP	40 mg/kg	Low	[68,72]
Urethane	IV/IP/Inhalation	800 mg/kg/1.4 g/kg/5%	Low	[73,94]
Enflurane	Inhalation	1–5%	Low	[37]
Propofol	IV	10 mg/kg	Low	[28]
Isoflurane + Pentobarbital	Inhalation + IV	Isoflurane: 1.5–2% Pentobarbital: 20 mg/kg/hr	Low	[30]
Chloroform	IP	3.5%	Low	[67]
ND	-	-	Moderate	[20,23,27,35,43,49,58,95,99,117,137,142]

Notes: Isoflurane was the most frequently used anesthetic. Considerable variability in dosing and reporting was observed. * Very high (*n* ≥ 30), high (*n* = 15–29), moderate (*n* = 5–14), and low (*n* < 5). Abbreviations: IP, intraperitoneal; IM, intramuscular; IV, intravenous; ND, not defined.

**Table 4:** Evaluation of infarct size outcomes in rat tifMCAO models.

Outcome	Method/Assay	Typical Reporting Format	Typical Assessment Window (Post-MCAO)	Key Methodological Note	Ref.
Infarct size/Absolute	TTC staining or histology (planimetric analysis) MRI (T2-weighted, DWI)	mm^3^	~24 h (standard) 3–72 h (acute) ≥7 d (chronic)	Overestimated without edema correction	[18,22,24,29,34,45,50,52,77,79,80,83,88,92,107,109,115,117,121,136,139]
Infarct size/Relative (Normalized to contralateral or ipsilateral hemisphere)		%	~24 h (standard) 3–72 h (acute) ≥7 d (chronic)	Multiple formulas Limited comparability	[18,25,28,36,37,40,43,46,47,48,50,51,52,53,54,56,58,59,64,65,67,70,71,74,78,79,81,83,85,87,88,92,94,95,96,101,103,104,105,107,108,109,114,115,116,121,127,129,130,131,134,141]
Edema-corrected infarct size (Swanson method)		% or mm^3^	~24 h (standard) 3–72 h (acute) ≥7 d (chronic)	Reduces swelling bias	[22,24,25,27,28,34,36,52,54,55,56,62,65,70,77,83,87,100,101,104,107,111,115,116,117,118,121,127,129,136,141]
Ischemic core/penumbra	MRI (DWI/PWI), histology	Volume or area	≤24 h	Definitions vary widely	[79,91,112]

Notes: Infarct volume is typically assessed at 24 h post-MCAO. Edema correction is recommended in acute infarct assessment to reduce overestimation. Abbreviations: DWI, diffusion-weighted imaging; MRI, magnetic resonance imaging; PWI, perfusion-weighted imaging.

**Table 5:** Brain edema assessment in rat tifMCAO models.

Outcome	Method/Assay	Typical Reporting Format	Typical Assessment Window (Post-MCAO)	Key Methodological Note	Ref.
Brain water content	Wet/dry method	% water content	24–72 h	Terminal No spatial resolution	[18,25,26,43,47,51,54,57,74,101,102,131,141]
Brain swelling	Hemispheric volume comparison	% swelling	24–72 h	Segmentation variability	[28,37,43,100,104,115,118]
MRI edema	T2-weighted imaging, ADC mapping, T1 mapping	Signal intensity/volume	6–72 h	Protocol variability	[81,139]

Notes: Brain edema is typically assessed between 24 and 72 h post-MCAO. The wet/dry method quantifies total water content, whereas MRI-based approaches enable spatial and longitudinal evaluation. Abbreviations: ADC, apparent diffusion coefficient; MRI, magnetic resonance imaging.

**Table 6:** Blood–brain barrier disruption in rat tifMCAO models.

Outcome	Method/Assay	Typical Reporting Format	Typical Assessment Window (Post-MCAO)	Key Methodological Note	Ref.
BBB permeability	Evans Blue extravasation	µg/g tissue or optical density	6–48 h	Dependent on dye circulation time, extraction method, and normalization	[26,37,43,63,100,101,110,121,131,138,141]
	Fluorescent tracer extravasation	Fluorescence intensity or tracer concentration	6–48 h	Strongly influenced by tracer size and molecular weight	[33,43,48,76,110]
	Contrast-enhanced MRI	Permeability index or signal enhancement	24–48 h	Protocol- and scanner-dependent Enables in vivo longitudinal assessment	[41,80,139]
BBB integrity/structural alteration	Tight-junction proteins and MMPs	Relative protein or mRNA expression	24–72 h	Indirect measure Does not quantify permeability	[27,37,43,58,95,101,110,131,138]
BBB transport/permeability kinetics	In situ perfusion/radiolabeled tracers	Uptake rate or permeability-surface area product	≤24 h	High physiological control Technically demanding	[100]

Notes: BBB permeability was commonly assessed between 6 and 48 h post-MCAO in the included studies. Evans Blue and fluorescent tracers assess macromolecular leakage, whereas contrast-enhanced MRI enables in vivo permeability assessment. Tight-junction proteins and MMP-related markers reflect molecular BBB alterations but do not directly quantify permeability. Abbreviations: BBB, blood–brain barrier; MMPs, matrix metalloproteinases; MRI, magnetic resonance imaging; mRNA, messenger RNA.

**Table 7:** Assessed molecular and peripheral biomarkers in rat tifMCAO models.

Sample	Biological Process/Outcome Class	Typical Assessment Window (Post-MCAO)	Ref.
Brain	Inflammatory	30 min–30 d	[19,20,21,27,32,41,43,44,47,51,53,54,56,57,58,73,74,75,76,78,79,81,83,85,86,87,90,93,94,95,96,99,102,105,107,111,112,113,115,116,120,123,124,126,131,132,134,135,138]
	Neurodegeneration/Neuronal injury	30 min–28 d	[21,27,28,30,32,39,49,53,54,55,61,62,78,79,83,87,96,98,102,106,107,112,114,115,116,123,125,126,127,129,132,135,136,140]
	Apoptotic	30 min–30 d	[18,20,32,37,42,47,56,60,65,73,74,78,79,81,83,94,96,99,102,106,115,119,120,121,124,132,134,135,136,137,138]
	Oxidative stress	30 min–14 d	[20,21,27,32,46,47,51,53,78,81,90,92,93,109,111,115,121,122,126,128,134]
	Gliosis (astrocytic response)	2 h–35 d	[18,38,44,49,62,68,78,81,83,87,91,116,120,125,131,132,135,136,137]
	Neuroinflammation (microglial activation)	12 h–35 d	[21,32,38,62,68,74,99,107,120,123,125,126,127]
	Histopathology/Tissue morphology	30 min–35 d	[25,32,38,47,49,78,115,124,125,132,137]
	Molecular BBB-related markers	2 h–30 d	[27,37,43,58,62,83,95,101,114,131,138]
	Angiogenesis	2 h–14 d	[44,47,62,93,96,99,110,137]
	Mitochondrial	4.5 h–14 d	[32,66,81,92,93,116,122]
	Autophagy	6 h–14 d	[25,71,112,116,130,136]
	Calcium signaling	30 min–14 d	[32,61,67,100,101]
	Neurotransmitter	30 min–14 d	[32,36,67,72,115]
	Neuroplasticity	24 h–28 d	[49,52,105,127]
	Myelin-related	24 h–28 d	[123,133,135]
	Coagulation	24 h–3 d	[27,58]
	Synaptic	24 h–14 d	[28,98]
	Electrophysiological	24 h–3 d	[104]
	Excitotoxicity	24 h–14 d	[36]
	Hemodynamic	24 h–3 d	[118]
	Hypoxia	6 h–24 h	[142]
Blood	Inflammatory	24 h–35 d	[31,46,47,57,82,85,86,96,97,99,141]
	Organ function (kidney)	24 h–5 d	[35,57,141]
	Organ function (liver)	6 h–5 d	[35,36]
	Angiogenesis	24 h	[57,110]
	Neuro-related circulating markers	24–25 h	[57,82]
	Neuroendocrine (HPA axis)	24 h–14 d	[85,86]
	Metabolic	24 h–72 h	[57,141]
	Oxidative stress and lipid peroxidation	24 h	[97]
	Apoptotic	25 h	[82]
	Glutamatergic activity	6 h–4 d	[36]
	Hypoxia	24 h	[57]
	Pain-related	24–72 h	[141]
Cecum/Colon	Gut microbiota	24 h–35 d	[31,69]
Urine/Kidney	Kidney function, hypoxia	6 h–24 h	[142]
Retina	Retinal neurodegeneration/Inflammation	24 h–7 d	[23]

Legends: Outcomes are grouped according to sample type and biological process. Considerable heterogeneity was observed in both the type of biomarkers assessed and the timing of evaluation across studies. Categories referring to specific cell types were integrated into broader biological processes for consistency. Abbreviations: d, days; h, hours; min, minutes.

**Table 8:** Neurobehavioral, cognitive, and physiological outcomes in rat tifMCAO models.

Domain	Outcome/Measurement	Typical Assessment Window (Post-MCAO)	Ref.
Neurological function	Neurological score (mNSS, Bederson, Garcia, Longa)	2 h–42 d	[19,20,22,25,27,28,29,34,36,37,39,43,44,46,47,48,49,50,51,52,53,54,56,57,58,59,61,62,64,65,67,71,73,74,75,78,79,80,83,85,86,90,92,93,94,97,99,100,101,102,103,104,105,106,107,110,111,112,113,114,115,116,119,120,121,122,124,126,127,129,130,131,132,133,135,138,141]
Motor function	Rotarod, beam walking, grip strength, foot-fault, cylinder test	4 h–42 d	[19,22,26,28,31,36,37,38,39,42,46,52,55,62,64,65,66,71,74,82,87,90,92,93,96,107,108,111,129,131,138]
Cognitive function	Spatial learning and memory (MWM, Barnes maze)	24 h–42 d	[26,27,42,74,103,110,119,129]
Affective behavior	Anxiety- and depression-like behavior (EPM, FST, sucrose preference)	24 h–35 d	[31,86]
Physiological parameters	Body weight changes	2 h–28 d	[19,22,44,52,66,80,85,86,124,141]

Notes: Outcomes are grouped by functional domain. Considerable heterogeneity was observed in both the type of tests used and the timing of the assessment, as well as in the assessment scoring systems applied in different studies. Abbreviations: d, days; EPM, elevated-plus maze; FST, forced swimming test; h, hours; mNSS, modified neurological severity score; MWM, Morris water maze.

**Table 9:** Reporting quality assessment of the included studies using the ARRIVE guidelines.

Final Grade	Number of References
High Quality	124
Moderate Quality	1
Low Quality	-

## Data Availability

No new data were created or analyzed in this study. Data sharing is not applicable to this article.

## Supplementary Materials

The following supporting information can be downloaded at: https://www.mdpi.com/article/10.3390/cimb48060632/s1.

## Abbreviations

The following abbreviations are used in this manuscript:

4-HNE	4-Hydroxynonenal
8-OHdG	8-Hydroxy-2′-deoxyguanosine
ADC	Apparent diffusion coefficient
ASC	Apoptosis-associated speck-like protein containing a CARD
BBB	Blood–brain barrier
BDNF	Brain-derived neurotrophic factor
BrdU	Bromodeoxyuridine
CAT	Catalase
d	Days
DCX	Doublecortin
DWI	Diffusion-weighted imaging
EPM	Elevated-plus maze
FST	Forced swimming test
GDNF	Glial cell line-derived neurotrophic factor
GFAP	Glial fibrillary acidic protein
GSH	Glutathione
GSH-Px	Glutathione peroxidase
h	Hours
Iba1	Ionized calcium-binding adaptor molecule 1
IM	Intramuscular
IP	Intraperitoneal
IV	Intravenous
LAMP1	Lysosome-associated membrane protein 1
LC3-II	Light chain 3 II
MAP2	Microtubule-associated protein 2
MBP	Myelin basic protein
MCAO	Middle Cerebral Artery Occlusion
MDA	Malondialdehyde
min	Minutes
mm	Millimeters
MMPs	Matrix metalloproteinases
mNSS	Modified neurological severity score
MRI	Magnetic resonance imaging
mRNA	Messenger RNA
MWM	Morris water maze
NeuN	Neuronal nuclei
ND	Not defined
NF-κB	Nuclear Factor kappa B
NSE	Neuron-specific enolase
PWI	Perfusion-weighted imaging
S100B	S100 calcium-binding protein B
SOD	Superoxide dismutase
T-AOC	Total antioxidant capacity
TGF-β	Transforming growth factor-beta
tifMCAO	Transient intraluminal filament middle cerebral artery occlusion
TNF-α	Tumor Necrosis Factor-alpha
VEGF-A	Vascular endothelial growth factor A
ZO-1	Zonula occludens 1
