# Supplementary material for: Transient Middle Cerebral Artery Occlusion in Rats as a Nonclinical Model of Ischemic Stroke: A Systematic Review
Source: Curr Issues Mol Biol. 2026 Jun 17;48(6):632. doi: 10.3390/cimb48060632 (PMC13298307; doi:10.3390/cimb48060632)
Supplement: Supplementary file 1 [file cimb-48-00632-s001.zip › cimb-4334328-supplementary.pdf]

Supplementary Material

Table S1: Comprehensive search expression applied in the biomedical electronic databases MEDLINE (Pubmed), Web of Science, and Scopus, on March 26th, 2025, including the records published between 2018 and 2025.

Database and Search Expressions	Records
**MEDLINE (PubMed)**	
("Infarction, Middle Cerebral Artery"[MeSH Terms]) OR (MCAO[Title/Abstract]) OR ("middle cerebral artery occlusion"[Title/Abstract]) OR ("middle cerebral artery infarction"[Title/Abstract]) OR ("Brain Ischemia"[MeSH Terms]) OR ("Brain Ischemia"[Title/Abstract]) OR ("Ischemic Stroke"[MeSH Terms]) OR ("Ischemic Stroke"[Title/Abstract]) OR ("ischemic brain injury"[Title/Abstract]) OR ("cerebral ischemic injury"[Title/Abstract]) AND (Intraluminal) OR (“Intraluminal Filament”) OR (“Intraluminal monofilament”) OR (monofilament) OR (Filament) OR (“Filament model”) OR (“intraluminal suture”) OR (“suture model”) OR (tifMCAO) OR (ifMCAO) OR (fMCAO) AND ("Animal Experimentation"[MeSH Terms]) OR ("Animal Experimentation"[Title/Abstract]) OR ("Laboratory Animal Model*"[Title/Abstract]) OR ("Experimental Animal Model*"[Title/Abstract]) OR ("Disease Models, Animal"[MeSH Terms]) OR ("Disease Animal Model*"[Title/Abstract]) OR ("Animal Disease Model*"[Title/Abstract]) OR ("preclinical stud*"[Title/Abstract]) OR ("pre-clinical stud*"[Title/Abstract]) OR ("nonclinical stud*"[Title/Abstract]) OR ("non-clinical stud*"[Title/Abstract]) OR ("in vivo"[Title/Abstract]) AND (Rats[MeSH Terms]) OR ("Rat*"[Title/Abstract])	74
**Scopus**	
TITLE-ABS-KEY("middle cerebral artery occlusion") OR TITLE-ABS-KEY(MCAO) OR TITLE-ABS-KEY("middle cerebral artery infarction") OR TITLE-ABS-KEY("Brain Ischemia") OR TITLE-ABS-KEY("Ischemic Stroke") OR TITLE-ABS-KEY("ischemic brain injury") OR TITLE-ABS-KEY("cerebral ischemic injury") AND ALL(Intraluminal) OR ALL(“Intraluminal Filament”) OR ALL(“Intraluminal monofilament”) OR ALL(monofilament) OR ALL(Filament) OR ALL(“Filament model”) OR ALL(“intraluminal suture”) OR ALL(“suture model”) OR ALL(tifMCAO) OR ALL(ifMCAO) OR ALL(fMCAO) AND TITLE-ABS-KEY("Animal Experimentation") OR TITLE-ABS-KEY("Laboratory Animal Model*") OR TITLE-ABS-KEY("Experimental Animal Model*") OR TITLE-ABS-KEY("Disease Animal Model*") OR TITLE-ABS-KEY("Animal Disease Model*") OR TITLE-ABS-KEY("preclinical stud*") OR TITLE-ABS-KEY("pre-clinical stud*") OR TITLE-ABS-KEY("nonclinical stud*") OR TITLE-ABS-KEY("non-clinical stud*") OR TITLE-ABS-KEY("in vivo") AND TITLE-ABS-KEY("rat") OR TITLE-ABS-KEY("rats")	146
**Web of Science**	
TI=("middle cerebral artery occlusion") OR AB=("middle cerebral artery occlusion") OR TI=(MCAO) OR AB=(MCAO) OR TI=("middle cerebral artery infarction") OR AB=("middle cerebral artery infarction") OR TI=("Brain Ischemia") OR AB=("Brain Ischemia") OR TI=("Ischemic Stroke") OR AB=("Ischemic Stroke") OR TI=("ischemic brain injury") OR AB=("ischemic brain injury") OR TI=("cerebral ischemic injury") OR AB=("cerebral ischemic injury") AND ALL=(Intraluminal) OR ALL=(“Intraluminal Filament”) OR ALL=(“Intraluminal monofilament”) OR	15

ALL=(monofilament) OR ALL=(Filament) OR ALL=(“Filament model”) OR ALL=(“intraluminal suture”) OR ALL=(“suture model”) OR ALL=(tifMCAO) OR ALL=(ifMCAO) OR ALL=(fMCAO) AND TI=("Animal Experimentation") OR AB=("Animal Experimentation") OR TI=("Laboratory Animal Model*") OR AB=("Laboratory Animal Model*") OR TI=("Experimental Animal Model*") OR AB=("Experimental Animal Model*") OR TI=("Disease Animal Model*") OR AB=("Disease Animal Model*") OR TI=("Animal Disease Model*") OR AB=("Animal Disease Model*") OR TI=("preclinical stud*") OR AB=("preclinical stud*") OR TI=("pre-clinical stud*") OR AB=("pre-clinical stud*") OR TI=("nonclinical stud*") OR AB=("nonclinical stud*") OR TI=("non-clinical stud*") OR AB=("non-clinical stud*") OR TI=("in vivo") OR AB=("in vivo")

Table S2: Animal-related parameters across the included studies.

Authors, Year [Reference]	Animal-related parameters				
	Strain	Sex	Age (weeks)	Weight (g)	Sample size (n)
Song M., 2022 [99]	Sprague-Dawley	Male	6	220-240	8
Yamasaki T., 2021 [123]		Male	6-7	190-230	25t
Ma J., 2018 [76]		Male	6-7	220-240	6
He Y., 2022 [48]		Male	6-7	200-300	40
Shu L., 2019 [98]		Male	6-7	220-240	ND
Dumot C., 2022 [38]		Male	6-8	199 ± 13	25t
Meng F., 2023 [78]		Male	6-8	ND	6
Zhang Q., 2024 [132]		Male	6-8	ND	5
Sun J., 2025 [101]		Male	7	260-280	ND
Candamo-Lourido M., 2024 [24]		Male	7-8	250-300	6
Dopico-López A., 2021 [36]		Male	7-8	250-300	103t
Wang Y., 2022 [114]		Male	7-8	235-250	12t
Li Q., 2020 [69]		Male	7-8	280-320	9
Wang H., 2024 [111]		Male	7-8	250-300	ND
Huang X., 2021 [54]		Male	7-10	ND	176t
Bai X., 2024 [20]		Male	8	180-200	32
Fu G., 2024 [42]		Male	8	300-340	5
Kwon J., 2019 [140]		Male	8	280-310	38t
Hong L., 2022 [49]		Male	8	230-300	120t
Lee S., 2019 [65]		Male	8	250-300	11
Huang S., 2018 [53]		Male	8	260-300	9
Yoon J., 2018 [125]		Male	8	280-300	8
Nakuluri K., 2019 [142]		Male	8	ND	6
Xu D., 2021 [120]		Male	8-9	250 ± 10	3
Zhu Y., 2021 [137]		Male	8-9	280-320	3
Gorenkova N., 2019 [44]		Male	8-9	240-290	24t
Yong L., 2023 [141]		Male	8-10	280-320	62
Wu C., 2022 [118]		Male	8-10	290-330	14
Yu H., 2019 [126]		Male	8-12	220-250	10
Wong A., 2023 [117]		Male	9-10	290-360	8t
Yang C., 2022 [124]		Male	9-11	260 ± 20	3
Verma M., 2021 [109]		Male	10-12	235-337<br>292-392	17<br>32
Franx B., 2021 [41]		Male	11-13	ND	19t
Mukda S., 2019 [81]		Male	12	366-390	4-5
Alam J., 2020 [19]		Male	12	328 ± 20	25
Stanton J., 2022 [100]		Male	12	200-250	6
Menzie-Suderam J., 2018 [79]		Male	16-18	260-300	20
Dandekar M., 2022 [31]		Male	Young Adult	250-270	12
Duan Y., 2020 [37]		Male	Adult	280-320	43
Lan J., 2018 [64]		Male	Adult	300-320	12
Zhai Z., 2018 [129]		Male	Adult	250-280	45t
Zhao H., 2022 [133]		Male	Adult	260-280	48
Tukhovskaya E., 2021 [108]		Male	Adult	320-380	9
Liu X., 2021 [74]		Male	Adult	280-300	6-8

Liu H., 2024 [71]	Sprague-Dawley	Male	Adult	250-280	6
Taboada-Rosell K., 2024 [103]		Male	Adult	280-310	26
Zhang L., 2024 [131]		Male	Adult	250-280	4-15
Zhang H., 2023 [130]		Male	Adult	300 ± 10	75t
Jia T., 2022 [56]		Male	Adult	240-260	6
Xu H., 2021 [122]		Male	Adult	250-280	9
Tanioka M., 2020 [105]		Male	Adult	260-300	310t
Sun K., 2022 [102]		Male	Adult	240-260	10
Pravalika K., 2019 [90]		Male	Adult	240-270	ND
Li L., 2018 [68]		Male	Adult	280-320	8
Nguyen H., 2019 [84]		Male	Adult	~250	6
Yu Q., 2019 [127]		Male	Adult	280-320	56
Xu H., 2018 [121]		Male	Adult	250-300	5
Zhao Y., 2020 [134]		Male	Adult	230 ± 20	8
Sarmah D., 2022 [93]		Male	Adult	240-270	10
Khelif Y., 2018 [62]		Male	ND	300-350	ND
Kuo D., 2024 [139]		Male	ND	250-300	20t
Desilles J., 2018 [33]		Male	ND	ND	ND
Huang S., 2018 [52]		Male	ND	250-320	8
Cheng Q., 2019 [27]		Male	ND	ND	ND
Wen M., 2019 [116]		Male	ND	250-280	13
Shi J., 2021 [97]		Male	ND	180-220	12
Chen G., 2018 [25]		Male	ND	250-280	12
Li Z., 2024 [136]		Male	ND	~320	29t
Liu J., 2019 [72]		Male	ND	250-270	18
Jiren Z., 2024 [59]		Male	ND	300-350	6
Morgan C., 2020 [80]		Male	ND	299 ± 30	15t
Tai S., 2022 [104]		Male	ND	240-290	20
Hsu H., 2024 [50]		Male	ND	250-300	ND
Termulaeva R., 2024 [106]		Male	ND	200-240	6
Wang Q., 2018 [113]		Male	ND	220-250	45t
Jiang W., 2020 [57]		Male	ND	220 ± 20	12
Shao Y., 2022 [95]		Male	ND	220-250	6
He J., 2020 [47]		Male	ND	250 ± 30	ND
Zuo W., 2018 [138]		Male	ND	260-280	ND
Tobin M., 2020 [107]		Female	14-16	225-275	3
Bake S., 2019 [21]		Female	40-48	325-350	5–9
Wang P., 2019 [112]		Female	72-80	375-450	8
Liu R., 2021 [73]		Female	Adult	250-300	24t
Biose I., 2022 [22]		Male	48-52	519-641	35
		Male	48-52	330–405	9
		Male	36-28	408–641	9
		Female	48-52	280–336	9
Plotnikov E., 2020 [88]		Male<br>Female	6-8	220-260	10
Dan L., 2019 [67]		Male<br>Female	ND	240-260	6 or 10
Xie Q., 2021 [119]		ND	7-8	250-300	9
Lu P., 2021 [75]		ND	ND	230-270	10
Zhao Z., 2021 [135]		ND	ND	240-270	6

Khaksar S., 2022 [60]	Wistar	Male	8-12	230-330	223t
Ferreira L., 2021 [40]		Male	10-12	300 ± 20	6
Ma S., 2019 [77]		Male	10-12	250-300	37
Onufriev M., 2022 [86]		Male	12	200-300	8(KM)<br>9(LM)
Onufriev M., 2021 [85]		Male	12	200–300	85t
Dimopoulos C., 2020 [35]		Male	12	390±5.5	10
Cipolla M., 2020 [30]		Male	15-25	ND	8
Gubskiy I., 2018 [45]		Male	Adult	250-300	89t
Abbasi Y., 2019 [18]		Male	Adult	280-300	12
Khaksar S., 2025 [61]		Male	Adult	250-350	135t
Diamanti S., 2023 [34]		Male	Adult	280 ± 5	45 (HDT15)<br>59 (FLAT)
Blixt F., 2019 [23]		Male	Adult	280-350	23
Hamadjija A., 2024 [46]		Male	Adult	220-250	6
Nazarian S., 2020 [83]		Male	Adult	280-300	12
Ramírez-Sánchez J., 2021 [92]		Male	Adult	250-285	ND
Ramírez-Sánchez J., 2019 [91]		Male	Adult	260-285	12
Nardai S., 2020 [82]		Male	ND	280 ± 20	8
Liebenstund L., 2021 [70]		Male	ND	349.3 ± 36.7	16
Kozler P., 2024 [63]		Male	ND	400	15t
Chernysheva G., 2024 [28]		Male	ND	250-280	15
Hu X., 2024 [51]		Male	ND	280-300	6
Jin R., 2022 [58]		Male	ND	280-320	ND
Gholami L., 2022 [43]		Male	ND	200-250	3-7
Pérez-Mato M., 2019 [87]		Male	ND	300 ± 25	48t
Semeleva E., 2020 [94]		Male	ND	250-300	5
Eldahshan W., 2019 [39]		Female	12-24	240-310	18 (1*)<br>ND (2*)<br>ND (3*)
Ingberg E., 2018 [55]		OVX Female	14	296 ± 22	20
Dergunova L., 2018 [32]		ND	ND	200-250	15
Vila E., 2019 [110]	Wistar-Kyoto	Male	14	ND	19
Choi S., 2018 [29]		Male	Adult	270-300	45t
Chen W., 2024 [26]	Lewis	Male	ND	280-300	10
Yueniwati Y., 2024 [128]	ND	Male	7-9	≥ 200	5
Li C., 2019 [66]	ND	Male	Adult	260-280	30t
Wei X., 2019 [115]	ND	Male	ND	ND	10
Shen D., 2023 [96]	ND	ND	ND	ND	10
Pochechuev M., 2022 [89]	ND	ND	ND	ND	ND

Abbreviations: Ct, control; FLAT, flat position; HDT15, Head down tilt 15°; KM, Koizumi Method; LM, Longa Method; min, minutes, ND, Not defined; OVX, ovariectomized; t, total.

Table S3. Ischemia monitoring across the included studies.

Systems/ Probes	Reference
PeriFlux system 5000	[22,24,72]
PeriFlux 5001 with PR407-1 probe	[108]
PeriFlux System 4001	[76]
Other fixed or straight-needle LDF probes	[23,31,65]
LDF (without specifying the exact configuration)	[21,25,27,30,34,48,50,52,53,55,64,66,67,70,79,82,84,90,93,98,100,104,109,110,113,116,121,129,137,138]
Magnetic Resonance Imaging	[24,29,36,38,41,45,56,62,80,81,107,139,140]
Frequency-domain near-infrared spectroscopy	[118]
Near-Infrared Spectroscopy	[117]
Positron Emission Tomography	[123]
Real-time intravital imaging coupled with laser	[33]
Laser Magnetic Flowmetry	[94]
Implantable fiber-optic probes combined with genetically encoded fluorescent sensors	[89]
MoorFLPI-2 speckle flow system	[115]

Table S4. Level of agreement between reviewers.

Screening phase - Rayyan					
		**Reviewer 2**		**Total**	***kappa* coefficient**
		**Included**	**Excluded**		
**Reviewer 1**	**Included**	133	15	148	**0.76**
	**Excluded**	5	52	57	
**Total**		138	67	205	

Risk of bias Assessment SYRCLE						
		**Reviewer 2**			**Total**	***kappa* coefficient**
		**Yes**	**Unclear**	**No**		
**Reviewer 1**	**Yes**	585	32	0	617	**0.889**
	**Unclear**	39	615	1	655	
	**No**	0	0	8	8	
**Total**		624	647	9	1280	

Reporting Quality Assessment ARRIVE						
		**Reviewer 2**			**Total**	***kappa* coefficient**
		**Yes**	**Unclear**	**No**		
**Reviewer 1**	**Yes**	2222	27	1	2250	**0.898**
	**Unclear**	46	275	0	321	
	**No**	1	1	115	117	
**Total**		2269	303	116	2688	

Reviewer 1: C.M., Reviewer 2: I.G.

Figure S1. Study-level risk of bias assessment of the included studies using the SYRCLE tool. Study references correspond to the reference numbers used in the main manuscript. Panels A–D correspond to sequential groups of included studies and are presented separately for readability.

Panel A

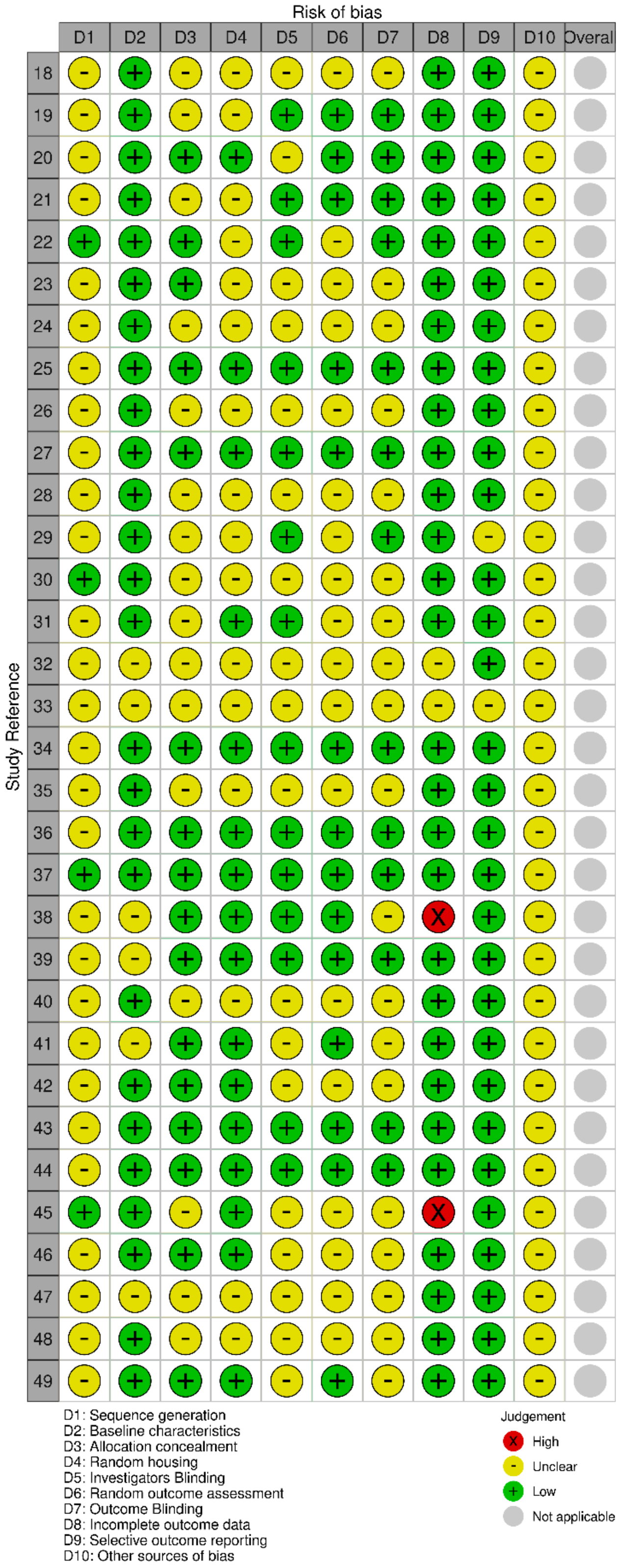

Panel B

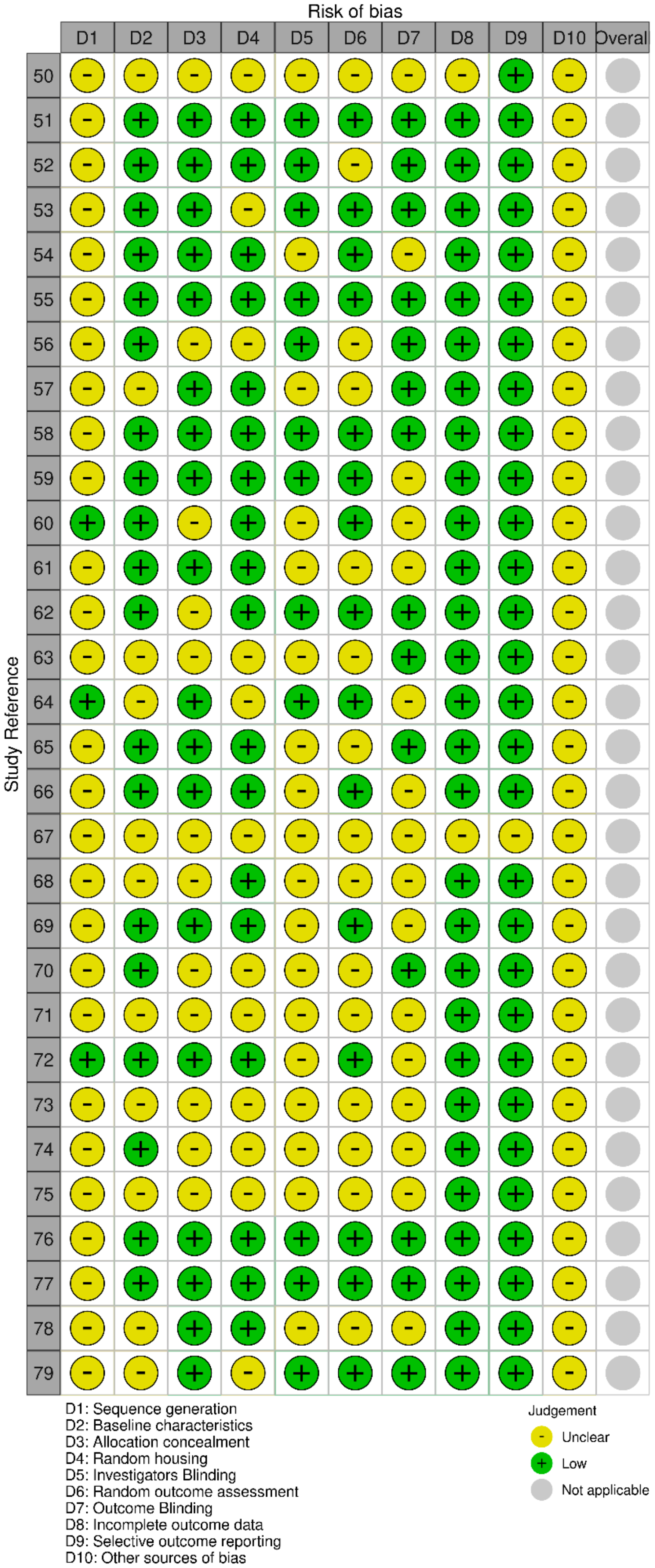
Risk of bias
D1
D2
D3
D4
D5
D6
D7
D8
D9
D10
Overal
Study Reference
50
51
52
53
54
55
56
57
58
59
60
61
62
63
64
65
66
67
68
69
70
71
72
73
74
75
76
77
78
79
D1: Sequence generation
D2: Baseline characteristics
D3: Allocation concealment
D4: Random housing
D5: Investigators Blinding
D6: Random outcome assessment
D7: Outcome Blinding
D8: Incomplete outcome data
D9: Selective outcome reporting
D10: Other sources of bias
Judgement
Unclear
Low
Not applicable

Panel C

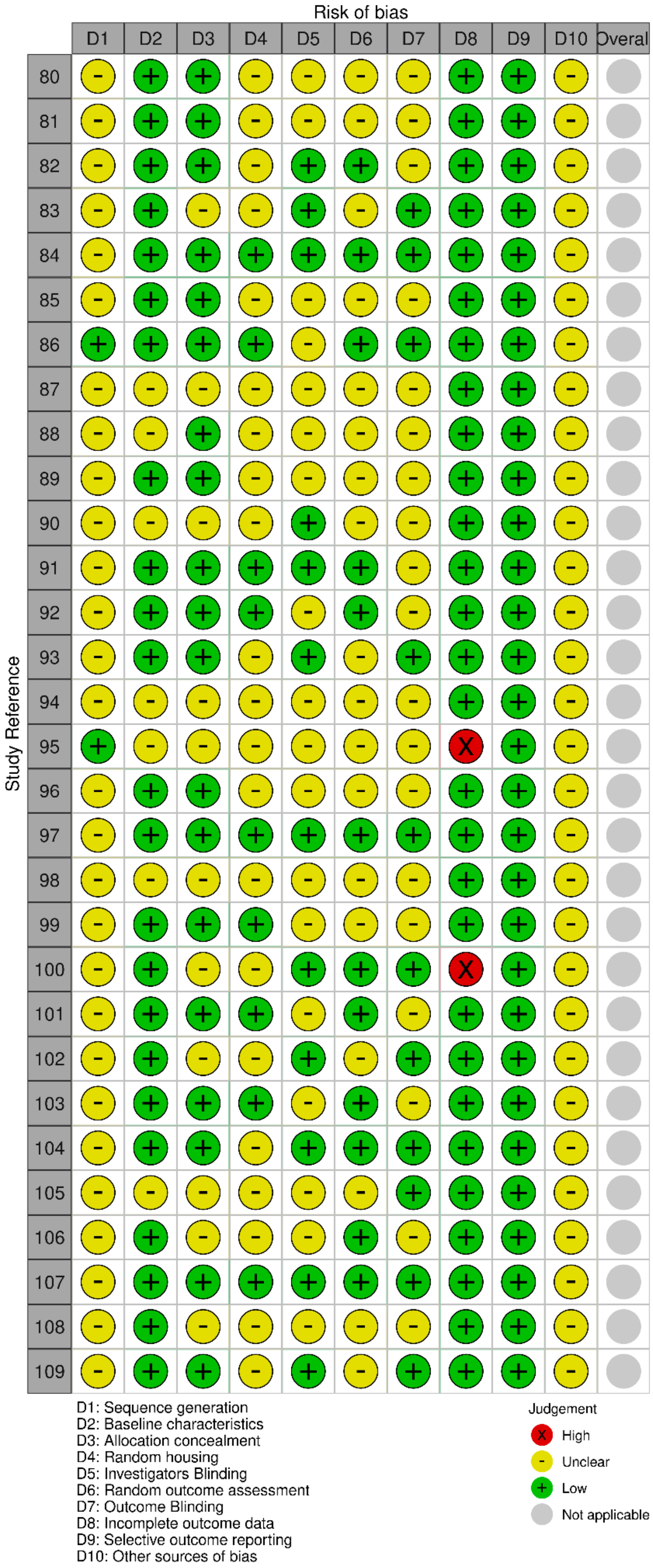
Risk of bias
D1
D2
D3
D4
D5
D6
D7
D8
D9
D10
Overall
Study Reference
80
81
82
83
84
85
86
87
88
89
90
91
92
93
94
95
96
97
98
99
100
101
102
103
104
105
106
107
108
109
D1: Sequence generation
D2: Baseline characteristics
D3: Allocation concealment
D4: Random housing
D5: Investigators Blinding
D6: Random outcome assessment
D7: Outcome Blinding
D8: Incomplete outcome data
D9: Selective outcome reporting
D10: Other sources of bias
Judgement
High
Unclear
Low
Not applicable

Panel D

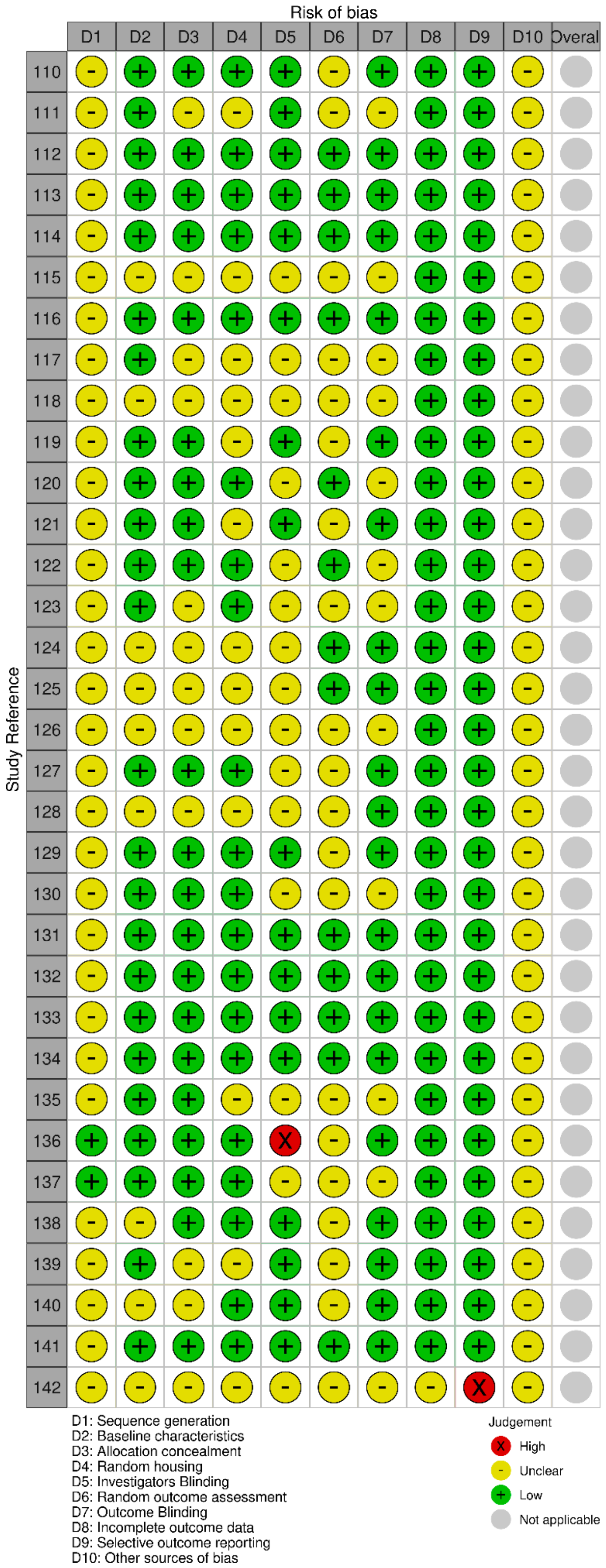
Risk of bias
D1
D2
D3
D4
D5
D6
D7
D8
D9
D10
Overall
Study Reference
110
111
112
113
114
115
116
117
118
119
120
121
122
123
124
125
126
127
128
129
130
131
132
133
134
135
136
137
138
139
140
141
142
D1: Sequence generation
D2: Baseline characteristics
D3: Allocation concealment
D4: Random housing
D5: Investigators Blinding
D6: Random outcome assessment
D7: Outcome Blinding
D8: Incomplete outcome data
D9: Selective outcome reporting
D10: Other sources of bias
Judgement
High
Unclear
Low
Not applicable
